# A Pragmatic Turn in the Study of Early Executive Functions by Object Use and Gestures. A Case Study from 8 to 17 Months of Age at a Nursery School

**DOI:** 10.1007/s12124-020-09578-5

**Published:** 2020-10-19

**Authors:** Cintia Rodríguez, Iván Moreno-Llanos

**Affiliations:** Department of Psychology, Autónoma University of Madrid, C/Iván Pavlov, n. 6, 28049 Madrid, Spain

**Keywords:** Executive functions, Own challenges, Private gestures, Uses of objects and instruments, Early education, early development

## Abstract

The two first years of life are critical in the development of Executive Functions (EF). However, very little is known about their early manifestations, how they develop, how they relate to other psychological constructions or the status of other people’s influence in this early development. The study of EFs has been carried out through standardised tasks, but some authors question their ecological validity and suggest an approach involving everyday situations and the challenges that children set for themselves. In this article we ***first*** review these issues in relation to the first manifestations of EFs. We ***secondly*** present a longitudinal case study at nursery school of a child between the ages of 8 and 17 months, considering the challenges and the means he employed in order to resolve them. We found that, from 8 months of age, the child gave himself challenges in relation to the functional uses of objects and instruments. He regulated his own behavior both through object and instrument uses and private gestures. He also involved the teacher at 17 months. This finding suggests that (1) the material world is particularly important in these early manifestations of EF, (2) teachers’ interventions are essential. Implications of the findings for early years education are discussed.

## Introduction

No-one doubts the importance of self-control of behaviour to cope with everyday life. As Luria said

Man not only does not react passively to arriving information, but creates *intentions*, forms *plans and programs* of this action, controls their execution, and *regulates* his behaviour in such a way that it conforms to his conscious activity, comparing the effects of these actions with the original intentions and correcting every error that he has made. (Luria 1974, p. 78, emphasis in the original)

The first manifestations of these cognitive control skills occur in the final third of the first year of life, when the child sets himself/herself challenges to try to resolve (Diamond 2006; Zelazo and Müller 2007). Even though the two first years of life are critical in the development of EFs (Kochanska et al. 2000), very little is yet known about what these early manifestations consist of, how they develop and how they relate to other psychological constructions. According to Marcovitch and Zelazo (2009) “relatively little is known about the *origins of executive function in infancy* and the way in which executive function *develops* during the first years of life” (p. 1; emphasis added). It may not help that various traditions in psychology which have enquired into executive functions have placed language in a hegemonic position – twice over: (1) the tests, their goals and the way to resolve them are communicated verbally; and (2) it is assumed that language is *the* instrument *par excellence* of self-regulation, both in cognitivist models with the phonological loop, through top-down processes (Baddeley 2007/2016; Tirapu et al. 2002), and in the sociocultural stances of Luria and Vygotsky with private speech (Winsler et al. 2009; Winsler et al. 2011). Despite the urgent need to understand how early manifestations of executive functions are produced, there are few studies of children under two years old. The most common approach, as presented in the next section, is to use simplified and adapted versions of *standardised tasks* designed for adults.

Albeit timidly, researchers are increasingly claiming that in order to study the first manifestations of executive functions (EFs), language should be disregarded, or at least left in the background (Neale et al. 2018), and the status of *self-directed gestures* and *action* should be seriously considered. Moreover, authors are increasingly questioning the *ecological validity* of standardised tests and suggesting that tasks should be more like everyday situations (Barker et al. 2014; White and Carlson 2016). Significant challenges that the child sets himself/herself in everyday life should be analysed, rather than simply the “challenges” set by the experimenter (Barker and Munakata 2015) in which doubt remains regarding whether or not they really are challenges for the child. Questions are also increasingly being raised regarding the usual segmentation of the processes involved in EFs (Chévalier 2010; Zelazo and Müller 2007). More than three decades ago, Kopp (1982) said, “scant attention has been paid to the developmental course of self-initiated regulation and behaviour” (p. 199).

Following some of these critics, in the first part of this paper we will review the state of the art of research in early Executive Functions. In the second part, we will present a longitudinal case study of a child between the ages of 8 and 17 months, considering the significant challenges and the means he discovered for himself to resolve them in classroom 0–1 at nursery school. The purpose is to introduce a pragmatic turn in the study of the first manifestations and early development of EFs, from the end of the first year of life.

To achieve this objective, the starting point is to identify what *challenges* children set *for themselves* in *everyday situations*. This involves considering the child as an *agent,* with his or her own initiative in relation to what goals to set and what means to employ to achieve them. Another aspect to highlight is the emphasis on the study of the *development* of *executive functioning*, both of *goals* and of the *means* employed. It involves analyzing the semiotic constructions from the end of the first year of life, both gestures and uses of objects and instruments (categories will be shown in Table 3). The analysis of the specific *materiality* involved is very relevant to understand the degree of complexity of the goals and means. As a consequence, language loses its hegemonic place as *the* instrument of cognitive control and evaluation in these early stages of development. This study also considers the educative influence of the teacher, both through her interventions and the materials made available to the children in the classroom. The underlying idea is that if children begin to intentionally control their own behavior by the end of the first year of life, it is because the other played some *guiding* role. A guide, the teacher, provides meaning and semiotic tools (gestures, uses of objects and instruments, language, etc.) that the child makes his/hers as an agent.

### EF Components and Standardised Tasks for Measuring them

There is a fairly general consensus on what the main *components* of EFs are and how to *measure* them. The most widely used method for measuring EF components consists of applying batteries of tasks with pre-established goals, from the second year of life (see in Zelazo and Müller 2007, p. 449–451, a detailed selection of tasks and the authors that inspired them; see also Diamond 2013).

The three core EFs (Chévalier 2010; Diamond 2013; Miyake et al. 2000) most commonly accepted are: (a) *Inhibitory control*, which implies being able to control one’s attention, behaviour, thoughts and/or emotions, inhibiting what is not relevant. In tasks that measure *inhibition*, children have to inhibit a preponderant response (a correct response, eating a tempting snack, etc.), which creates conflict with the child’s everyday experience (Carlson 2005; Carlson and Moses 2001). Some very common tasks are the *Day-Night Task*, the *Delay-of-Gratification Task* and the *Stroop Task* (Bernier et al. 2010). (b) *Cognitive flexibility*, which is the ability to change flexibly between tasks or strategies, to change perspectives interpersonally, or to change how to think about something. To measure *flexibility,* the tasks require that once the child has provided a correct response following a given criterion (such as colour), the criterion is changed and the child must respond according to a different one (shape), and so on successively (see in Cragg and Chévalier 2012, p. 215, the best known paradigms in cognitive flexibility with children and adults). Some of the most commonly used tasks are the *Wisconsin Card Sorting Test* and the *Dimensional Change Card Sort* (Carlson 2005; Frye et al. 1995; Zelazo et al. 2003). Diamond (2013) notes that most 3-year-olds succeed in maintaining an initial sorting dimension, but fail at switching dimension in the second phase. (c) *Working memory updating*, which involves holding information in mind and mentally working with this information when it is no longer perceptually present. There seem to be two types of working memory: verbal and visual-spatial. To measure *working memory*, the child has to remember two rules, e.g., for stimulus 1, press on the same side as the stimulus; for stimulus 2, press on the side opposite the stimulus. Another method is to have the child repeat a series of meaningless numbers, words or letters presented by the experimenter. Some examples are *The Dots Task*, *The Digit Recall*, *Word Recall* and *Nonword Recall* (Gathercole et al. 2004).

Sometimes, a fourth component is added, (d) *Goal-setting*, which includes initiative, conceptual reasoning, planning and organising strategies (Anderson 2002, see also Lozano Gutiérrez and Ostrosky 2011). Goal-setting is a complex process which involves determining needs, knowing what one wants and what one is capable of doing (Barroso and León-Carrión 2002). From a problem-solving framework, Zelazo et al. (1997) include problem representation, planning, execution and evaluation. Other authors include more components (Tirapu-Ustárroz et al. 2017), as well as hot EFs, related to emotions (Zelazo and Müller 2007). According to García-Madruga et al. (2016) “Apart from the core and higher order cognitive EFs, there is another executive function clearly involved in an individual’s action: the emotional control of behavior” (p. 4).

Some studies on the emergence of EFs during the second year of life provide good illustrations of the use of this type of standardised tasks. For instance, Miller and Marcovitch (2015) conducted a longitudinal study with children at 14 and 18 months of age in relation to language and joint attention. They used the following tasks: (a) *A-not-B* with five hiding locations and a 10 s delay (a more difficult version of Piaget’s A-not-B task); (b) *The Forbidden Toy Task*: every time the children attempt to pick up a train moving along a rail, while they are performing another less interesting task, they are reminded that “they can’t play with that toy now”; (c) *The Three Boxes*: children have to retrieve three rattles that “make noise” from three different boxes, inhibiting previous correct responses; and (d) *The Imitation Sorting Task*: the experimenter classifies the objects, “the frog goes in this basket” and encourages the child to imitate him/her, “now you try”. Miller and Marcovitch conclude that children’s performance was initially poor, and found little evidence for internal consistency across EF measures.

Bernier et al. (2010) conducted a study with 80 mother-infant dyads at 12, 15, 18 and 26 months in the home. They stress the role of the early parent-child relationship in the development and differences in children’s EF. They used two tasks, (a) *Hide the Pots*: a sticker is hidden under a pot, and the pot is hidden under a blanket. The child has to hold the location of the sticker in memory, remove the blanket and reach for the pot where the sticker was hidden, and (b) *Categorisation*: children are taught a categorisation rule. Three baby animals are sorted in the ‘baby box’ whereas three adult animals are placed into the ‘mommy box’. The experimenter then asks the child to sort the six remaining ones. Many children placed all six animals together in one box. According to Bernier et al. (2010) “more research is needed before one can confidently conclude that either of our 18-month tasks can be used by itself as an indicator of early EF” (p. 336). As in the study by Miller and Marcovitch (2015), here too, little evidence for internal consistency across EF measures was found. Indeed, internal consistency also presents difficulties in adults. Miyake et al. (2000) suggest that it may be because people adopt different strategies on different occasions, even within a session, when performing these tasks.

#### Standardized Tasks: Some Difficulties for Studying Early EFs

The standardised tasks present a number of difficulties from the point of view of studying EFs: (1) perhaps one of the keys to the difficulty found in these studies lies in the paradox of resorting to language for studying early executive functions (Basilio and Rodríguez 2017; Neale et al. 2018; Rodríguez et al. 2017a, 2017b; Rodríguez et al. 2020). In most of the tasks, the rules are communicated verbally. The experimenter *says what* should be done, *when* and *how* (it is assumed that the child *understands what is said*). (2) Functional aspects of the objects are usually absent. Segmented “physical” attributes are often used, such as colour, shape or size, which have little or nothing to do with the functional rules of objects in everyday life. (3) Inhibition is usually evaluated through situations *contrary to children’s everyday experience*. This does not cause major problems in 5-year-olds or adults, who already have plenty of experience with the use of language in simulated situations (Barthélémy-Musso et al. 2013). However, in younger children, it alters their conventional and functional everyday relationship with the world. This implies considerable difficulty since from the end of the first year, children are very actively constructing social rules about how to relate functionally to the world, and adults play a very active part in the inception of these rules (Rodríguez and Moro 1999; Alessandroni and Rodríguez 2020). Yet in the inhibition tasks, an adult asks the child to violate these rules after having made them! A good example is the *Day-Night Task*, where children have to say “day” when a picture of the moon is presented and “night” with the sun. There must be other ways to study inhibition at these ages without violating everyday rules. (4) Another feature of the studies on EF is that, regardless of the number of components involved, they are usually analysed in a *segmented* manner*.* Some critical voices, such as Zelazo and Müller (2007) react against these EFs as a homunculus (by simply listing) and highlight the complexity of their functionally organised elements. For example, Cragg and Chévalier (2012) consider that cognitive flexibility in children and adults is not limited to a mere change in tasks: “flexibility appears to be underpinned by an array of processes: some being related to task decision/goal setting and others to shifting task sets *per se*; some being intentional in nature and others being automatic” (p. 226). The same is true for inhibition. Finally, the tasks are impure because it is difficult for a given task to measure one single EF (Chévalier 2010; Miyake et al. 2000).

At this point let us now step back several decades to consider Luria and Piaget’s positions. They are particularly relevant today. What is common in both is their interest in studying psychological *processes* through a *constructivist* and *developmental* perspective. Both studied *early development* of *cognitive control* at a *microgenetic level*. That makes an important difference in relation to the use of standardized tasks for adults and simplified for children. This will be shown in the next section.

#### Early Manifestations of EFs: Luria, Piaget and the Paradox of the A-Not-B Error

Luria undoubtedly pioneered the study of executive functions (although the term was introduced by Muriel Lezak in Lezak 1982) based on his research during World War II with persons with injuries to the frontal lobes. Afflictions included deficits in initiative, motivation, goal setting, action plans and self-control of behaviour (Tirapu et al. 2002). Luria suggests that the brain is composed of non-modular interactive functional systems with the frontal and prefrontal cortex being in charge of programming by setting goals, regulating the course of action and consciously evaluating one’s own behaviour. Luria says that since the beginning of his career he was fascinated by the function of language in the “formation and regulation of human activity” (Luria 1979, p. 104). In a paper published in Luria 1959, *The Directive Function of Speech in Development and Dissolution*, Luria describes the broad lines of how to study executive functions in children from 12 months. In this article, not often cited in the literature on early executive functions, Luria analyses “the role of the word in the organisation of mental life” (ibid., p. 341), and how the directive, significant, generalising function of words is formed during child development. Upon investigating how the self-regulatory function of words arises in early development, Luria describes, *in fact*, previous forms of external regulation through action and use of objects (for a review of these studies, see Rodríguez et al. 2020. Nevertheless, his focus was always language. At first, the language directed to the child by others (external regulation) and later on, the child’s self-directed language.

Before referring to Piaget, we should mention briefly the influence on Vygotsky (1934/1987) and Luria of Piaget’s (1923/1976) studies on *egocentric speech*. The interpretation of the same phenomenon – the child talks to himself/herself – is diametrically opposed between them. For Piaget, it is residual, reflecting egocentric thought; the child talks to himself/herself aloud because he/she is not yet sufficiently socialised. Vygotsky (1934/1987) and Luria however, assign to egocentric speech (*private speech,* as it is known today) a central role as an instrument of self-regulation of the child’s own behaviour; its root is in social language. Beyond these differences, it is relevant to recall that due to his premature death in 1934, Vygotsky did not learn of Piaget’s studies on sensorimotor development published from 1936. Be that as it may, other than exceptions, for the sociocultural tradition language was and still is *the* instrument for self-regulation (Bronckart 2013).

Let us now consider Piaget’s ideas. Part of Piaget’s work during the 30s and 40s on sensorimotor development has had great influence on the study of self-regulation and early executive functions. He situates *action* as the origin of cognitive control from the second half of the first year of life with the first goal-directed activities. Adele Diamond says:

According to Piaget, the *first signs of what we could today call EF are evident by 8-9 months of age* (SM Stage 4) […]. When infants reach for a desired object, it is hard to tell whether the external stimulus elicited an automatic reach or the intention was internally generated. However, when an infant searches for an object that is not visible, or *acts on an object of no particular interest in order to obtain a desired object*, then Piaget was willing to infer that *intentionality* was present and the action sequence have been truly *goal-directed* (i.e., executively controlled). (2006, p. 71, emphasis added)

Capilla, too, considers that Piaget’s sensorimotor development stage IV can be considered “the most rudimentary stage of *planning* and *problem solving*, which are considered to be *executive functions*”. This require “an internal representation of the goals and means to achieve them as well as their *manipulation* and *monitorisation*” (Capilla 2007, page. 33, emphasis added, translated by us).

No doubt then. Diamond and Capilla agree about taking stage IV as the beginning of EFs (a term never used by Piaget himself). In fact, one of Piaget’s (1936/1965) main goals was to understand how children *intentionally* control their own behaviour – what happens when they distinguish between means and ends. It is the goal they set themselves that directs their course of action, even before using tools. This is consistent with his definition of intelligence: “The act of intelligence properly so called develops in that way, to the extent that it is differentiation of the secondary circular reaction and involves to a higher degree the “reversal“ in the consciousness which constitutes intention […]” (p. 213, Piaget 1936/1965. This reversal (*renversement*) between causal order and mental order means that on the mental plane, the goal comes first, while on the causal plane, the means need to be activated first (for a discussion, see Rodríguez 2006). This involves momentary *inhibition* of the *goal*, in order to activate the *means* that will enable it to be attained. It also requires certain *flexibility* because the means may vary. The means depend on the goal set by the child, the child’s level of development, or the available means. It also involves *direction* of behavior because it is the goal that *guides* it (not chance, as in previous stages). All of this is in tune (at least in early manifestations during the first year of life) with Marsh and Iran-Nejad who equated intelligence with self-regulation: “intelligence is ‘the capacity for self-regulation […]’” (1992, p. 330, as cited in Demetriou 2000).

From 4 or 5 months, infants already know themselves to be intentional *agents*, when, upon performing secondary circular reactions, they ultimately understand that the rattle rattled *because* they shook it (see, in Piaget 1936/1965, observations 94–119 on the development of this type of behaviour). A paradigmatic example of “intermediate” conduct is the challenge set by Laurent, aged 6 months and one day, as described in observation 120:

Laurent tries to grasp a big piece of paper that I offer him and finally place on the hood of his bassinet (and on the string connecting the hood with the handle of the bassinet). Laurent begins by stretching out his hand; then as soon as the object is placed, reacts as he always does in the presence of distant objectives: he shakes himself, waves his arms, etc. The desire to grasp the paper seems to inspire such reactions, as I regulated it by removing the objective from the hood for a few seconds in order to move it progressively closer and farther away. It is when the paper seems inaccessible to the hand alone that Laurent shakes himself. After having behaved thus for a moment, *he seems to look for the string hanging from the hood, then pulls it harder and harder while staring at the paper*. At the moment when this is ready to fall off the hood, Laurent lets go the string and reaches toward the objective of which he immediately takes possession. Several successive attempts have yielded the same result. (Piaget 1936/1965, p. 214, emphasis ours)

Piaget also admits his doubts as to whether Laurent is displaying “anticipated” early actions typical of stage IV with means-ends distinction, which is an important novelty in psychological development. He says:

It goes without saying that it cannot be demonstrated that Laurent pulled the string *in order to* grasp the paper, but the whole behaviour pattern *gave me the impression* of being performed with this end in view and of being perfectly coordinated. If such is the case, it can be asserted that the schema “pulling the string” has momentarily served as *means* to attain the *end* assigned by the schema “grasping the objective”. This of course does not mean that Laurent has foreseen the object’s fall, nor that he has conceived of the string as its extension: He has simply utilized a familiar schema with a *new intention*, and *this is what characterizes the behaviour patterns of the fourth stage*. (Piaget 1936/1965, p. 214, emphasis ours)

Later on, in stage V, towards 11 or 12 months, children begin to use tools. The novelty in relation to previous stages is that a mediating instrument, rather than the hand, acts upon the objective. This adds major complexity to planning and execution. Here we should mention pioneer studies by Köhler 1921/1989; see also Friedrich 2013) with chimpanzees using tools, which directly inspired this part of Piaget’s work (for a discussion, see Rodríguez 2006).

At the end of the sensorimotor period, stage VI, children find solutions by “mental combination” (nowadays we would say top-down processes) before acting directly on the environment. The novelty is that children stop their action in order to think and find a solution. When one of Piaget’s daughters thinks about how to remove a chain from a closed matchbox and opens her mouth representing a cavity, there is “plastic reflection” (see obs. 180): “due to inability to think out the situation in words or clear visual images she uses a simple motor indication as ‘signifier’ or symbol” […] (Piaget 1936/1965, p. 338), to think about how to open the box and find a solution (for a discussion, see Rodríguez 2009).

To conclude, we should highlight that Piaget links the origin of cognitive regulation to *action* and interiorization of action schemata (Piaget 1936/1965. This marks a clear difference with the status of language in the cognitive or sociocultural traditions. Piaget also offers a developmental perspective, with longitudinal studies. His analyses of the processes involved in the objectives and the increasingly complex means used by his children (with objects, instruments or through symbols) are paradigmatic, and an example of the best literature on the origin and development of cognitive control from the second half of the first year. Careful reading of *The origins of intelligence in children* (Piaget 1936/1965 [*La naissance de l’intelligence chez l’enfant*], in particular from stage III, can still shed much light on understanding the first manifestations of executive functions and self-regulation through action in everyday contexts. These advantages, however, are clouded by Piaget’s lack of interest in communicative and cultural influences from others. Piaget’s child uses his/her own resources, a product of his/her solitary action, past and present, to self-regulate. In relation to the origins of self-regulation, Luria’s and Vygotsky’s cultural framework offer a complementary perspective to Piaget’s.

If one considers the reception of Piaget’s ideas in the EFs literature, there is the paradox that his work on the birth of intelligence - with the ingredients of long processes of construction concerning planning, goal-directed behaviour set by the child himself/herself, flexibility to attain a goal according to the means used, inhibition of distracting stimuli, evaluation of what has been done, the child’s increasing certainty that he/she can do it, etc., − has gone relatively unnoticed. Piaget entered the literature on EF basically through the A-not-B error about the permanence of the object (an object-searching error, often made by 8–12 months olds). Infants will look for an object where they have most often found it - location A - rather than where they last saw it hidden - location B. The A-not-B error has been reinterpreted and widely *transformed* in a *standardised task* in the literature on cognitive development in general (see Bremner 2017 for a review), or to evaluate early EFs (Cuevas et al. 2018; Diamond 1985, 2006; Marcovitch and Zelazo 2009; Smith et al. 1999). A classic example is the work of Diamond (1985) during the second half of the first year. She introduced variable delays from the time she hid the object until the child was allowed to search for it (the father/mother held the child’s hands). She found that children who committed the A-not-B error stopped erring if the delay was reduced to 2–3 s. “So difficult is the task at longer delays that infants ‘give up’ or become so frustrated that their performance does not improve even when easier trials, at shorter delays are presented later” (p. 878–879). She concludes that performance of the task improves with age, suggesting development of the ability to use recall to guide action.

From the dynamic systems tradition, Smith et al. (1999) also used A-not-B error. They manipulated different aspects, such as the visual properties of the hiding locations, their transparency and number, or the delay between hiding and search. They made a critical review of its significance in terms of the dynamics of the here and now relative to the experience of the goal-directed reaching, including the previous experience, distancing themselves from Piaget:

For Piaget, success in searching for a displaced hidden object indexed an ‘object concept’, an enduring knowledge structure about the spatial and temporal constancies of objects. Our studies, as well as scores of others, have demonstrated, however, that performance in this task itself is far too fluid, too context dependent, too easily derailed by even seemingly minor changes in conditions to be a reliable indicator of some enduring structure of mind. (*ibid*., p. 258)

Finally, we will refer to a Marcovitch and Zelazo (2009) study where they developed a sophisticated model based on the A-not-B error. They consider that the search for hidden objects contains all the elements of a typical measure of executive function. “To solve the task, children must represent the object’s current location, keep this information in mind, and then use it to guide their search. If they err, they must detect their errors and correct them” (p. 2). There is *inhibition* (not to continue searching in A), *flexibility* or shifting (to change and search in B) and *updating* the *working memory* (the object is no longer in A, but in B). Like Smith et al. (1999), they too distance themselves from Piaget, though in the opposite direction. They base their position on the habituation studies by Baillargeon and DeVosb, according to which children have object permanence from 3½ months, so that “the A-not-B error does not reflect a deficit in object permanence understanding, but rather an inability to control motor behaviour effectively” (2009, p. 3).

As said previously, the transformation of the A-Not-B error in a standardized task differs from Piaget’s theory. For him it was part of a long process followed by children in their construction of the object as permanent through his own action. The A-not-B error has somehow become “the tree” hiding “the forest” of cognitive control construction, which was so subtly analysed by Piaget during the two first years of life.

In the next section some studies about early manifestations of EFs with a focus on action and gestures will be discussed.

#### Action and Gestures in the Study of Early EFs

In the past decade, an increasing number of authors have argued that the first manifestations of EFs can be found in *actions* and *gestures*. This involves two clear advantages: (1) there is no need to pass through the filter of the experimenter’s language; and (2) it is based on development because infants interact with objects from 4 or 5 months (before 3 months if helped by an adult, see Moreno-Núñez et al. 2017; Rodríguez and Moro 2008), and then produce other-directed gestures at about 8 months (Cameron-Faulkner et al. 2015; Dimitrova and Moro 2013).

Neale et al. 2018; see also McClelland et al. 2019) explicitly state that it is necessary to step away from language as a central tool in the tasks given to children, making other methods of evaluation available. They propose the Grasping Task, an ‘object-based task’ which “reduces the *semiotic complexity* of the rules and instructions that infants are expected to follow” (p. 2, emphasis in the original). They conducted a longitudinal study with 36 children aged 12, 18 and 24 months in interaction with their mothers. Here, the object “speaks”, simplifying the task. A spoon laden with food is placed in front of the child in alternate orientations to measure its ability to inhibit the preferred hand or the previously used hand. They found the Grasping Task predicts, for instance, the delay task performance (it also measures inhibition) at 24 months of age. According to Neale et al. different toys might require different degrees of expertise. For instance, using a toy hammer is more difficult than a spoon at 18 months. They conclude that more tasks for infants should be developed where rules are not communicated through language but through “a familiar test object” (p. 9).

Sastre-Riba et al. (2015) also studied executive abilities through action. They filmed four groups of children aged 15 months – children with typical development, Down’s syndrome, hypothyroidism, and low birth weight – in spontaneous activity with figures placed on a magnetic surface. Possible actions were: cover/uncover, place on top/remove, pull, hit, group, align, stack, connect/separate, place and put away. They focused on inhibition and cognitive flexibility. They found that very soon there were differences between groups. Children with typical development attained the best executive functioning. In another study, Sastre-Riba (2009) presents the differences between typical and moderately premature infants without neurological problems, from 18 months, although early social interaction fulfils an important function by modulating the neurobiological basis (Sastre-Riba et al. 2007). They concluded that executive functions vary according to age and development type although further longitudinal studies are required to ascertain whether these differences are maintained throughout development.

McGuigan and Núñez (2006) also studied early development of EFs through action focussing in inhibition and working memory. Children aged 18 to 24 months performed four detour reaching tasks to take toy animals out of a transparent box. The experimenter provided a model of the correct task solution for each condition. They found that children could take 4 animals out of the box without difficulty by pulling a lever or removing a plastic clip before pulling the lever, without acting directly on the toy animal, both when it was visible to them or when the box was opaque. However, they found it very difficult to open the box in an arbitrary means-action task, where they had to pick up a telephone located on top of it or pick up the telephone and pull the lever. There was one exception, when the experimenter accompanied her demonstration with language providing meaning to the action: *Look! Mr. Cow is sleeping inside his house today *How will we wake him up so he can come out and play? *Look! Here is a telephone, let’s telephone Mr. Cow. The authors claim that this proves that the child is active from the start in the development of EFs. In any case, it is interesting that children accept pulling a lever in order to open a box, but that they resist an arbitrary task - there is no relationship between picking up telephones and opening boxes in everyday life.

We will conclude this section about actions with the longitudinal studies by Cerchiaro (2014) and Cerchiaro and Puche-Navarro (2014). From the perspective of dynamic systems theory, they show how children aged 15 to 26 months are very active. After putting a ball into the top of a multilevel structure, they have to then activate a device to remove the ball. The children self-regulate, reorganise their actions, make postural adjustments to increase or reduce distance from the device, coordinate bimanually or repeatedly rectify the procedures employed.

If we consider *gestures*, there is plenty of literature on their cognitive status. The influential works by Goldin-Meadow highlight that gestures play a part in learning and problem solving (e.g., see in Goldin-Meadow et al. 2012, “move gestures” involving mental rotation with children; see also Schwartz and Black 1996). Gestures are also considered a learning resource in second language acquisition (Gullberg et al. 2010). Spatial gestures can be different in different cultures (Dasen et al. 2009; Wilkins 2003). Adults also produce gestures to accompany speech or thought. Thus, according to McNeill, gestures and speech form a “multimodal unit that is considered as language itself” (McNeill 2000, p. 9). Research with infants has focused on other-directed gestures and their role in the origin and development of language (see Murillo and Belinchón 2012).

Kita et al. (2017), move away from the gesture-language approach. They argue that gestures play a central role in human cognition and share some properties with practical actions. Gesture is a “representational use of the general-purpose action generation system, which also generates practical action” (p. 246). They focus on representational gestures with a “self-oriented cognitive function”. They propose the *gesture-for-conceptualisation hypothesis*: “gesture activates, manipulates, packages and explores spatio-motoric information for the purposes of speaking and thinking” (p. 246). The importance of *private gestures* (self-directed gestures with a self-regulation function) in relation to early forms of cognitive self-regulation is being increasingly recognised (see review in Kuvalja et al. 2013; and Table 1)'. A classic example is self-directed pointing, identified by Bates et al. (1979), when, based on Piaget’s sensorimotor stage V, they studied the origin of intentional communication. It appears before other-directed pointing – see also Carpendale and Carpendale (2010). From a sociocultural standpoint, Delgado et al. (2009, 2011) consider that private pointing gestures serve to focus the child’s own attention and may be precursors of private speech. Pea (1980) refers to the self-directed use of the social gesture of negation with a self-prohibition function. Symbolic and aesthetic self-directed gestures have also been identified (Español 2006).

**Table 1:** *Self-directed and private gestures (ostensive, pointing, symbolic) in early development with self-regulation function*

	Gestures			Development		Months	N/Design	Studies
	O	P	S	Typ	DS	of age	L / T	
Self-directed and Private gestures	x			x		10–12	20/L	Cameron-Faulkner et al. (2015)
		x		x		2–12	3/L	Bates et al. (1975)
		x		x		6–14	1/L	Carpendale and Carpendale (2010)
		x		x		8–24	2/L	Delgado et al. (2010)
		x		x		10–16	27/T	Español and Rivière (2000)
		x		x		12–73		Delgado et al. (2009)
		x		x		24–48	39 + 39 T	Delgado et al. (2011)
			x	x		5–28	22/L	Vallotton (2008)
			x	x		11–36	103/L	Goodwyn et al. (2000)
			x	x		12–24	2/L	Español (2001)
			x	x		14–34	40/L	Özçalişkan et al. (2014)
			x	x		18–36	40/L	Pea (1980)
	x	x		x		14–18	47/L	Miller and Marcovitch (2015)
Self-directed and Private gestures when using objects and instruments	x	x	x	x		8–11	1/L	Rodríguez et al. (2017a, 2017b)
	x			x		8–16	6/L	Moro et al. (2015)
	x			x		8–20	2/L	Moro (2016)
	x	x			x	12–18	1/L	Rodríguez and Palacios (2007)
	x			x		9–78	1/L	Ishiguro (2016)
	x			x		11–15	4/L	Basilio and Rodríguez (2011); Whitebread and Basilio (2012)
	x	x	x	x		14–18	16/L	Basilio and Rodríguez (2017)
	x			x		13	1/L	Moro and Rodríguez (2005)
	x			x		25–61	58/L	Tapparel (2015)
	x	x	x	x		4–18	8/L	Guevara et al. (2020)

Note: **Gestures**. *O =* Ostensive. *P =* Pointing. *S =* Symbolic; **Development**. *Typ =* Typical. *DS =* Down syndrome; **Design**. *L =* Longitudinal. *T* = Cross-sectional

In the followings sections we will return to self-directed gestures with a function of self-regulation in everyday life involving challenges.

#### Ecological Validity: Significant Problems in Everyday Life and the Influence of Others

An unresolved issue in EFs is knowing *when children set themselves challenges* in everyday life, *what those challenges consist of* and *what children do to resolve them*. Increasingly, it is claimed that subjects should be studied using longitudinal designs in real time while resolving significant problems (Moro 2012; Müller and Kerns 2015; Whitebread and Basilio 2012; see also Rader and Zukow-Goldring 2010). In standardised tasks, children are told what to do, and when and how to do it. However, “it is less clear how children’s experiences relate to their development of more *self-directed executive functioning*, where they must determine, *on their own, goal-directed actions* to carry out and *when*” (Barker et al. 2014 emphasis added, p. 3; see also Barker and Munakata 2015). In everyday life, “children are often required to apply rules that do not specify a concrete response” (ibid., p. 243).

According to Elkhonon Goldberg (2001), a student of Luria’s, we live in an ambiguous world: “Most real-life situations are inherently ambiguous. The answer is hidden, and so is the question” (pp. 77–78). He refers to the crucial difference between a typical memory experiment and the way in which memory is used in real life. It illustrates our purpose very well:

In real life, I have to make the decision what to remember. In a typical memory experiment the decision is made for me by the examiner: ‘Listen to these words and remember them’. By shifting the decision-making process from the individual to the examiner, we remove the role of the frontal lobes and the memory task is no longer a working memory task. Most real-life acts of recall involve working memory and the frontal lobes, but most procedures used in memory research and to examine patients with memory disorders do not. (Goldberg 2001, p. 73)

Moreover, it is difficult to talk of inhibition or flexibility without describing the *specific* contents at stake: what is inhibited or what flexibility refers to (Baker et al. 2010). When ecological validity increases, for example, in situations of symbolic play, with distancing strategies (*What would Batman do*?), the results in 5-year-olds improve noticeably (White and Carlson 2016).

The ecological validity of the tests is also of interest in evaluating, for instance, brain damage (Bombín-González et al. 2014), or autism (Acero et al. 2017; Kenworthy et al. 2008). Tirapu et al. (2002) highlight the difference between the structured laboratory situation and real life in neurological exploration. In the assessment process the examiner provides plans and initiates activities, often becoming the “frontal lobes” of the patient or research participant (Stuss and Alexander 2000, p. 290). In fact, in his Lezak 1982 paper, Lezak said the following about the use of neurological or psychological examination with damaged patients:

“[It is] very difficult if not impossible, to observe some of the most important executive functions, such as initiation of complex goal-directed activity, planning such activity, or carrying out one’s plans. The examiner who wants to observe these aspects of executive behavior is in a logically absurd position, for by its very nature the examination places the subject within a structured situation in which *the examiner dictates what the subject is to do, with what and when* […] (pp. 283 emphasis added).

Lezak’ words are particularly relevant for our purpose here in relation to the lack of ecological validity of standardized tasks used with children where the objectives are defined by the experimenter.

#### Private Gestures when Using Objects and Instruments that Involve a Challenge: Research in the Home

As discussed previously, the role of private gestures in cognitive self-regulation is becoming consolidated. Several longitudinal studies conducted in the home from the end of the first year show that when the challenges consist of using *objects and instruments with everyday functions,* children employ *private gestures* to self-regulate (see Table 1, above). What are these gestures? What is their *semiotic complexity?* From *when* are they produced? What is their *function*? (see Table 3 for a definition of gestures).

Those who claim that cognitive processes are anchored in communication are gaining strength. Parents provide support to the child’s autonomy, which affects the early performance of EFs (Bernier et al. 2010; Brinck and Liljenfors 2013; Moriguchi 2014). In a study with infants aged 10, 13 and 16 months, Moro (2012) shows how planning of goal-directed activity arises from situations of adult-infant-object interaction, where the adult initially introduces the infant to the action plan. In a beautiful observation, Moro shows how, after failing to place the head of a wooden cat on a post a 16-month-old performs a *self-directed (private) ostensive gesture*. This gesture implies stopping his action directed towards a goal and the beginning of a reflective activity with the objet itself. According to Moro, “a reorientation of the action takes place: first it was directed towards the goal, now the action is directed toward oneself” (p. 12 translated by us). The adult who is watching the scene shows the child the hole that will enable the wooden head to be placed on the post. It is not infrequent for adults to intervene on a child’s *precise* difficulty thanks to the fact that the private ostensive gestures are “*exteriorised* consciousness”. Reflective.

In another longitudinal study, a Swiss 13-month-old in the final session made several *private ostensive gestures* on being unable to use an object according to its function (Moro and Rodríguez 2005). An 18-month-old girl with Down’s syndrome (Rodríguez and Palacios 2007), in addition to private ostensive gestures, made private pointing gestures to help herself use a challenging object. Basilio and Rodríguez (2011) investigated how children from 11 to 15 months old used a hammering toy when they tried to put three balls into a box through three holes. Children produced *private ostensive gestures* to help themselves grasp the hammer in a way that would make it easier to hit the balls, or they presented the balls to themselves before placing them in one of the holes. Children also produced *vocalisations* in a similar way as adults use language (monitoring and evaluation functions). One of the girls, 15 months old, could not place the ball in the box, so she directed her hand with the hammer toward her father saying “eeh” asking for help, *but not giving him the control of the situation*. The girl indicated to her father, *what* to do, *when* and *how*. This is why it was considered self-regulation. In another longitudinal study (Basilio and Rodríguez 2017), children aged 14, 16 and 18 months performed private gestures, especially *ostensive gestures*, though they also helped themselves with pointing gestures and symbolic gestures to plan, monitor, control or evaluate their own action.

These studies show that children self-regulate with private gestures when they have difficulties in using everyday objects and instruments. Here are the ingredients of cognitive self-regulation and executive functions. *Private gestures combine flexibly* and creatively to achieve a goal: the functional use of the object or instrument, which is public, and therefore guarantees objectivity. They knew what the finishing point was, but had to *adjust how* to reach it, *inhibit* the irrelevant actions and act according to the objectives. The children tried to do what any adult would do with the objects. What adults had not explicitly taught them to do was to make private gestures! Moro et al. (2015) claim that “ostensive gestures involving an object that infants address to themselves are of special interest for studying the *link* between *consciousness* and *social practices* grounded in the *material world* before language” (p. 150, emphasis added).

#### Research at Nursery School

If social situations affect the learner’s self-regulation processes in primary school (Zimmerman 2000; Pintrich 2000; a review of models of self-regulated learning in Panadero 2017), they probably also do at nursery school. According to McClelland and Wanless, “in recent years, children’s self-regulation has emerged as a crucial area of interest in the education of young children” (McClelland and Wanless 2015, p. 609). In addition, there is evidence that low-income pre-schoolers tend to lag behind their higher-income peers in EF that may support the development of their academic skills (Nayfeld et al. 2013, p. 81). Improving self-regulation through appropriate early education seems to be particularly beneficial for children from disadvantaged backgrounds (Carboni et al. 2019). Teachers can play an important role in helping children regulate their behaviour and thinking in everyday experiences (Howard and Melhuish 2017). Tapparel (2015) conducted a study over one academic year, at a Centre de Vie Enfantine (Lausanne), on top-down processes in 3 to 4-year-olds during a painting activity with the teacher. Carr et al. (2017) analyse the spontaneous behaviour at school of 3 to 5-year-olds, in playscape environment when they face difficulties, or in interaction with a classmate (see also, Pyle and Danniels 2017, about play based learning pedagogies for kindergarten teachers, and Kangas et al. 2015).

Inspired by Vygotsky’s ideas on symbolic play as a promoter of self-regulation in early childhood, several studies have been carried out in nursery schools. For instance, Elias and Berk (2002) studied children 3–4 years-old. They found that complex sociodramatic play (not just any sociodramatic play) predicted development of self-regulation during clean-up periods in the preschool classrooms. This effect of play on self-regulation was particularly strong for highly impulsive children. Similarly, Bodrova et al. (2013) emphasize the importance of play in the development of self-regulation. They introduce the concept of “mature make-believe play” in order to measure “levels” of play. They express concern that 7 year-olds exhibit self-regulation levels more like those of the five-year-old children of the 1940s: they are not able to control their physical actions in following the directions of an adult. The authors attribute this phenomenon to the decline of both play quality and quantity that preschools and kindergartens now provide (p. 117). They also propose that more ecologically valid classroom-based observations are needed. This could provide valuable information about the development of play and the dynamics of its effects on self-regulation.

In another study with children 2–5 years old, Kroll (2017) found that teachers considered play to be central to the development of self-regulation. Play was used to learn about the world, for problem-solving (children begin talking to themselves) and as a way to regulate their emotions (see also Fleer et al. 2020, on how executive functions are promoted when children and teachers play together in collective imaginary situations).

#### Research at Nursery School with Children 0–2 Years Old

Despite the fact that the prefrontal regions of the cortex show more active development from 6 to 12 months (Capilla 2007; see also Nelson 2017; and Rivera et al. 2017, on neural plasticity and development), and that promoting autonomy is one of the primary objectives of the syllabus in infant education (Goble 2016), very little research has been conducted in the classroom with children in the first two years of life.

In an interesting study at a day-care centre in Japan, Ishiguro (2016) analyses microgenetically how a 15-month-old child learns to eat with a spoon when interacting with his teacher. The focus here is the transition from the child needing assistance to the moment he is capable of self-regulation. Ishiguro highlights different strategies: direct assistance (the child is fed), indirect (the spoon is given to the child) and semi-direct (his hand is guided). The novelty at 15 months is that the teacher now encourages self-regulation in the child: the teacher sequentially places the food so that it is accessible. Her concern is not limited to ensuring the child eats, in which case direct assistance would have sufficed. Rather, the teacher alternates with indirect assistance to encourage self-regulation. Ishiguro highlights the cultural consideration of food in Japan at these ages, which is included in the school curriculum as “Shokuiku”, education about food and nutrition.

In a case study (Rodríguez et al. 2017b), a child aged 11 months and 9 days eats dessert with a spoon after a laborious process of self-regulation based on a challenge set by his teacher at the beginning of the meal. He makes *private gestures* (ostensive, pointing and symbolic gestures) and displays *protocanonical uses* that are still ineffective for eating, but serve as an intermediate step until he uses the spoon for its purpose. It may be said that “the neural pathways that children employ … to bring a spoon from a bowl to the mouth will be strengthened with opportunity for practice” (Brock 2016, pp. 162–163). These outcomes suggest that these executive functions begin before the first year in nursery school in significant everyday situations.

Signs of self-directed speech using the Baby Sign Program at a nursery school have also been identified (Vallotton 2008). Another longitudinal study conducted in classroom 0–1 with 4 - to 12-month-olds (Guevara et al. 2020) found that it was not until 8 months of age that infants began to direct gestures to others, especially to their teacher. Between 4 and 8 months, gestures were self-directed. The most frequent gestures and the only ones performed by *all* participants were (i) *self-directed ostensions,* when children present an object to themselves in a contemplative way, exploring it, in the absence of later conventional uses or other challenges; and (ii) *private ostensive* gestures, which are more complex and were produced to self-regulate in a problem-solving context as they were followed by conventional uses of objects or instruments representing a challenge (Table 3 shows different private gestures). Even if *self-directed ostensions* are not gestures of self-regulation, given that they do not appear in relation to an explicit difficulty, they could nevertheless be precursors of more complex *private ostensive gestures*. Further research is required to establish the possible relationship between *self-directed* ostensive gestures and *private* ostensive gestures.

In relation to attention, children are usually considered to have a limited attention span, about 1 min on average for children aged 8 to 15 months (Toddler Attention Span: How long should they be able to focus? 2013). However, in a study with 5 to 13-month-olds in classroom 0–1 at nursery school during reading activity (in which they clearly show interest), the mean percentage of time for which the group of infants watched the teacher’s reading attentively was: 87% (3:25 min) in recording T1; 90% (2:32 min) in T2; 77% (3:19 min) in T3; and 85% (4:06 min) in T4 (Contin and Rodríguez 2020). The results suggest that infants could be using inhibitory control to remain focused on the book reading, ignoring potential distractors such as other infants or proximal objects.

These results, obtained in significant situations in the classroom, seem very promising for understanding executive abilities when the children *set themselves challenges* and the *means* to resolve them. The first axis that clearly appears is (1) self-directed *gestures* to control their own behaviour. Children use them to “tell themselves” many things when they are in significant challenging situations: “it’s not like this”, “I have to change it”, “I have to adjust this or that”, “I’m hungry”, “I did that really well”, or “help me please”. Systematic enquiry should be conducted into types of gestures produced when children resolve their daily challenges. (2) Another basic setting for the first forms of executive control, though less frequent in the literature, is the *pragmatic of action with objects and instruments.* Many of the challenges that children set themselves in everyday situations at nursery school consist of *how to do* something, how to use an object or instrument that presents difficulties and how to *modify* its uses if they are not sufficiently precise or do not match the challenges that they themselves set. Less efficacious uses become springboards enabling access to other more complex and efficacious ones. Since object and use do not coincide (Moro and Rodríguez 2005), knowing what *types of uses* infants perform (see Table 2; for a review, see Rodríguez et al. 2018), with what *objectives* and *how they evolve* during the first two years can potentially be very fruitful for drawing a map of self-regulation. All this, which is part of early socio-cognitive development, could shed much light on early EFs.

**Table 2:**
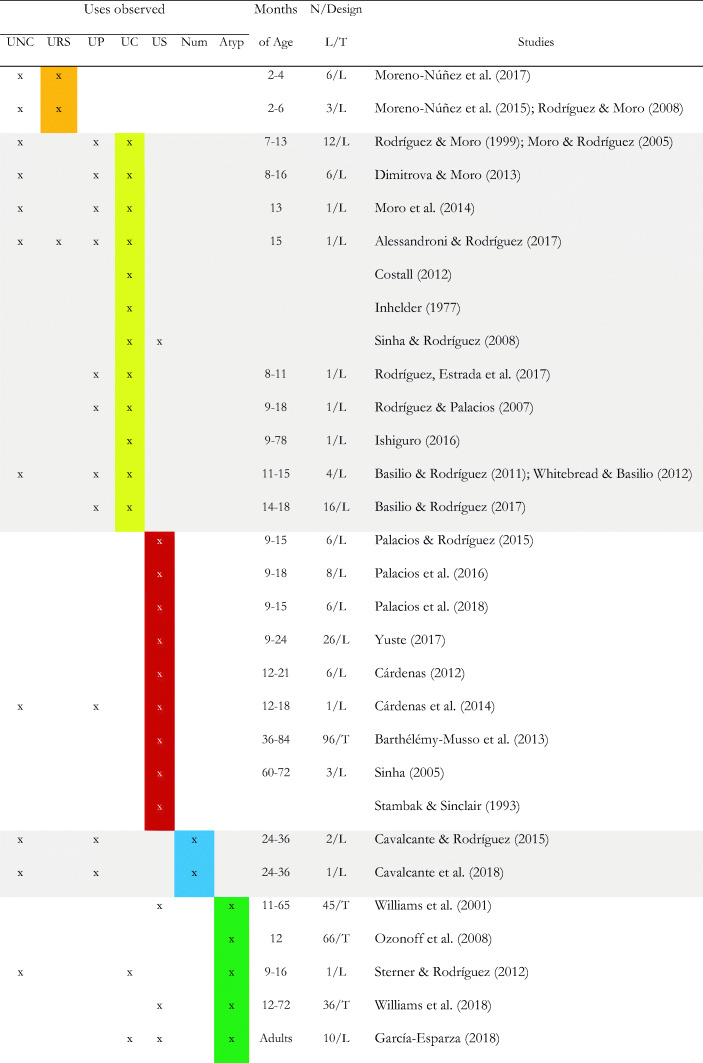
*Types of uses of objects and instruments by infants from 2 months. Highlighted uses studied*

Note: **Uses**. *UNC =* Non-Canonical. *URS =* Rhythmic-Sonorous. *UP =* Protocanonical. *UC =* Canonical. *US =* Symbolic. *Num =* Numerical. *Atyp =* Atypical; **Design**. *L:* Longitudinal. *T:* Cross-sectional

#### Case Study at the Nursery School

The case study presented here addresses the following four questions: (1) does the child set his own challenges (goals) from the end of the first year of life in everyday situations in classroom 0–1? (2) If so, what do the *goals* consist of and how do they *develop*? (3) What *means* does he employ to resolve his own challenges (achieve his goals) and how do they *develop*? (4) What is the role of potential *educational action* by the teacher in this important developmental achievement?

In order to answer these questions, we will identify (1) the challenging *goal* that the child sets *himself* as a component of EF and *within* it analyse (2) cognitive *flexibility*, (3) *inhibition,* (4) *attention* and (5) (when it takes place) *planning*.

## Method

### Participants

Even though all eight infants in the 0–1 classroom set themselves goals involving challenges, we initially selected the older children: three boys, Ils, I, and A, and one girl, P. After selecting the most significant sequences, we decided to conduct a case study with Ils. In contrast to the other infants, he was able to sit up without difficulty (Franchak 2018) from T_1,_ at which time he already set himself clear, complex goals. We identified and analysed sequences in which Ils set himself clear goals involving challenges from T_1_ (8 months and 7 days) to T_10_ (17 months). The teacher (hereinafter M) was included according to her participation in the course of Ils’ goal-directed activity.

### Procedure

Once a month, from October (T_1_) to July (T_10_), we videoed the morning activities in classroom 0–1, including the meal, at the nursery school “La Cigüeña María” (Madrid). We videoed the *educational situations* (see definition in Moro and Rickenmann 2004; Tapparel 2015, although the ages of the infants were younger than in the papers cited) planned by M and the teaching team. We never suggested any activity or change. This was a longitudinal, non-participant, natural observational study (León and Montero 2015).

We used a JVC-HD Everio video camera and a tripod. The experimenter stood behind it and endeavoured to remain as unobtrusive as possible. The filming angle was wide enough to include both M (except if she occasionally left the room) and the infants. If any of the infants went outside the viewing angle, videoing continued, including the largest number of children possible.

We viewed all the videos (T_1_-T_10_) of morning activities, except the meal (for a case study of the meal at this school, see Rodríguez et al. 2017b) and T_7_ (because Ils did not go to school on that day). We identified the *significant sequences* for Ils, which involved cognitive control of his own behaviour, whenever they related to the materials included in the educational situation proposed by M. We disregarded actions directed at external goals. Sequences were required to meet the following 5 criteria:

1. THE CHILD SETS HIMSELF THE GOAL. The child decides *what* significant goal to set, *when,* and *how fast* to execute it (there are no strict time limits). Due to its difficulty, the goal involves a challenge. If M specifically takes the initiative, the child must then appropriate the goal. Being an everyday classroom situation, the goals may vary according to available materiality. Since goal-directed behaviours do not form part of a predetermined “task”, they are to be identified through the child’s persistent action aimed at achieving a difficult goal.
2. ATTENTION TO THE OBJECTIVE. The child himself always determined where to direct his/her attention. Being an everyday situation, *Attention ON* moments clearly directed to the objective were identified, including attention to: (a) the objects and instruments that form part of the activity related to the goal, (b) the action itself, when the child looks with interest at his own use, gesture or the effects of the use performed, (c) M, when she intervenes to encourage, praise, evaluate, etc. what the child is doing, or when the child involves her in his own goal. *Attention OFF* was identified when the child deviates from the goal. These are moments of distraction, directing attention to other events (see in Goldberg 2001, the comparison between gibbons and dogs regarding return of attention to the task initiated after moments of distraction). An alternation takes place often between ON and OFF and ON again.
3. COGNITIVE FLEXIBILITY TO ATTAIN THE GOAL. Cognitive flexibility is shown through uses and gestures. The infant determines *which objects to use, how* and *when* to use them in a *functional and significative* way according to his *GOAL*. This decision involves being able to “navigate” through different uses, whether they are of the same or different semiotic level. The same is true of gestures. The child determines *what gestures* to produce*, how and when* to produce them in a *functional and significative* manner according to the GOAL. Flexibility is also shown through the *direction of behaviour*, such that one use may be self-directed – “eating with a spoon” – or other-directed – “feeding M” – in situations where the child himself maintains control of the situation. The same happens with gestures.
4. INHIBITION OF ATTENTION AND/OR BEHAVIOURS THAT DEVIATE FROM THE GOAL. (a) Inhibition of *Attention*: when the child inhibits attention OFF (which deviates from any of the components involved in attaining the goal) and activates attention ON regarding attainment of the goal. (b) Inhibition of *Uses of objects and instruments*: when the child inhibits uses of objects/instruments not relevant to the goal, e.g., non-canonical uses, in favour of relevant cultural uses in order to attain the goal.
5. PLANNING. Planning is shown when the child organises the material *before* performing the uses conducive to the goal set. We did not consider planning to be essential to the selection of sequences. It was only identified in isolated instances.

Because it was an everyday situation, we required the sequence to last at least 60 s. We considered that shorter times (classical EF tasks take a few seconds) would make it difficult to identify episodes according to the above criteria.

### Coding

We transcribed the selected sequences using the software Elan (v. 4.9.2, 2016; Lausberg and Sloetjes 2009) and encoded them following the procedure described in Rodríguez and Moro (1999) (see Table 3). We identified the following general categories in the child: (1) *Attention* (2) *Uses of objects and instruments* (3) *Gestures* (4) *Vocalisations-Speech.* We identified the *communicative direction* of gestures (Basilio and Rodríguez 2011; Basilio and Rodríguez 2017) and uses: (a) *self-directed* (private) (b) *directed to M*, as long as the child maintains control of the situation. In M we identified: (1) *Uses of objects and instruments* (2) *Gestures* and (3) *Speech.* For each sequence, we prepared specific categories according to the Goals that Ils set himself in T_1_, T_6_ and T_10_.

**Table 3:** *Categories. Ils’s self-regulation processes: Attention, Uses of objects and instruments, Gestures, and Vocalisations. M’s educational action: uses, gestures and speech*

Child (N)	
1. *Attention*	
*Teacher* AM	Looks at M or at M’s action
*Objective* AO	Looks at objective or means to reach it
*Own use* AU	Looks attentively at use of the object being employed
*2. Uses of Objects/Instruments*	
*Non-canonical uses* UNC	Non-specific uses, e.g., sucking objects or instruments
*Hints of Non-canonical uses UNCH*	The non-canonical use is not completed, e.g., opens mouth but does not suck object.
*Rhythmic-sonorous uses* URS	Using an object or instrument in a rhythmic-sonorous manner
*Rhythmic-sonorous canonical uses* UCRS	Using an object or musical instrument according to its function, e.g., shaking a bell
*Protocanonical uses* UP	Using an object or instrument in a manner close to, though not fully according to, its functional use
*Canonical uses* UC	Using an object or instrument according to its function
*Symbolic uses* US	Using an object or instrument to pretend to do something, e.g., “eating” with an empty spoon
*3. Gestures*	
*Other-directed*	
*Ostensive*	Gives or shows an object to M
*Pointing-touching*	Points-touches the referent for M
*Symbolic*	Represents an absent referent for M
*Self-directed*	
*Reaching*	Tries to reach an object with some difficulty
*Private ostensive*	Shows himself an object before using it for its function
*Private pointing*	Points for himself by touching the referent before using it for its function
*Private symbolic*	Represents an absent referent; e.g., clapping as the end of an own action
*4. Vocalisations /Speech*	Vocalisations and/or recognisable words
Teacher (M)	
*1.Uses of Objects and Instruments*	
*Distant rhythmic-sonorous demonstration*	M makes the object sound for Ils
*Distant symbolic demonstration*	M performs a symbolic use for Ils
*Immediate symbolic demonstration*	M involves Ils in a symbolic use
*2.Gestures*	
*Ostensive*	Gives or shows an object to Ils
*Placing*	Places an object or instrument near Ils
*Pointing*	Points to an object or image
*Symbolic*	Represents an absent reference
*3. Speech*	M directs speech to Ils

The inter-judge agreement Kappa index (León and Montero 2015) consisted of 50% of the time for each significant sequence. It was determined by comparing the encoding done by a second observer trained in the categories. The Kappa index found for sequence analyses was 0.82 (T10), 0.92 (T6 and T8a) and 1 (T1, T1b, T4, T8b). For the different categories, Attention obtained an index of 1, Uses of objects 0.95; Gestures 0.87; and M’s educational action 1. All indices are *substantial* or *Almost perfect* (Landis and Koch 1977).

In order to represent how Ils self-regulates, we identified manifestations of attention, flexibility, inhibition, and planning. (1) For *attention*, we counted total Attention ON time, which means the time spent by Ils paying attention to M, to his own objective and to his own uses. (2) For *flexibility,* we looked at how often Ils changed (a) from one type of functional use to another, (b) from a functional use to a gesture, or vice versa, and (c) from one type of gesture to another, as long as they were directed to his objective. (3) For *inhibition* of *distractors*, we looked at how often Ils changed from Attention OFF to ON. For *inhibition* of *uses*, we identified how often Ils changed from a non-relevant use (mainly UNC and UNCH) to a functional use or a goal-directed gesture. (4) For *planning*, we looked at the frequency of preparation behaviours performed by Ils in a sequence (T10).

## Results

Below, we present: (1) the *selected sequences* that meet the criteria for executive control (see above); (2) *Attention* ON/OFF of the selected sequences; (3) *Microgenetic Analysis* of three paradigmatic sequences (T_1_, T_6_ and T_10_) that show Ils’s executive control process and M’s intervention.

### Sequences Selected for Analysis of Executive Control

**Fig. 1:**
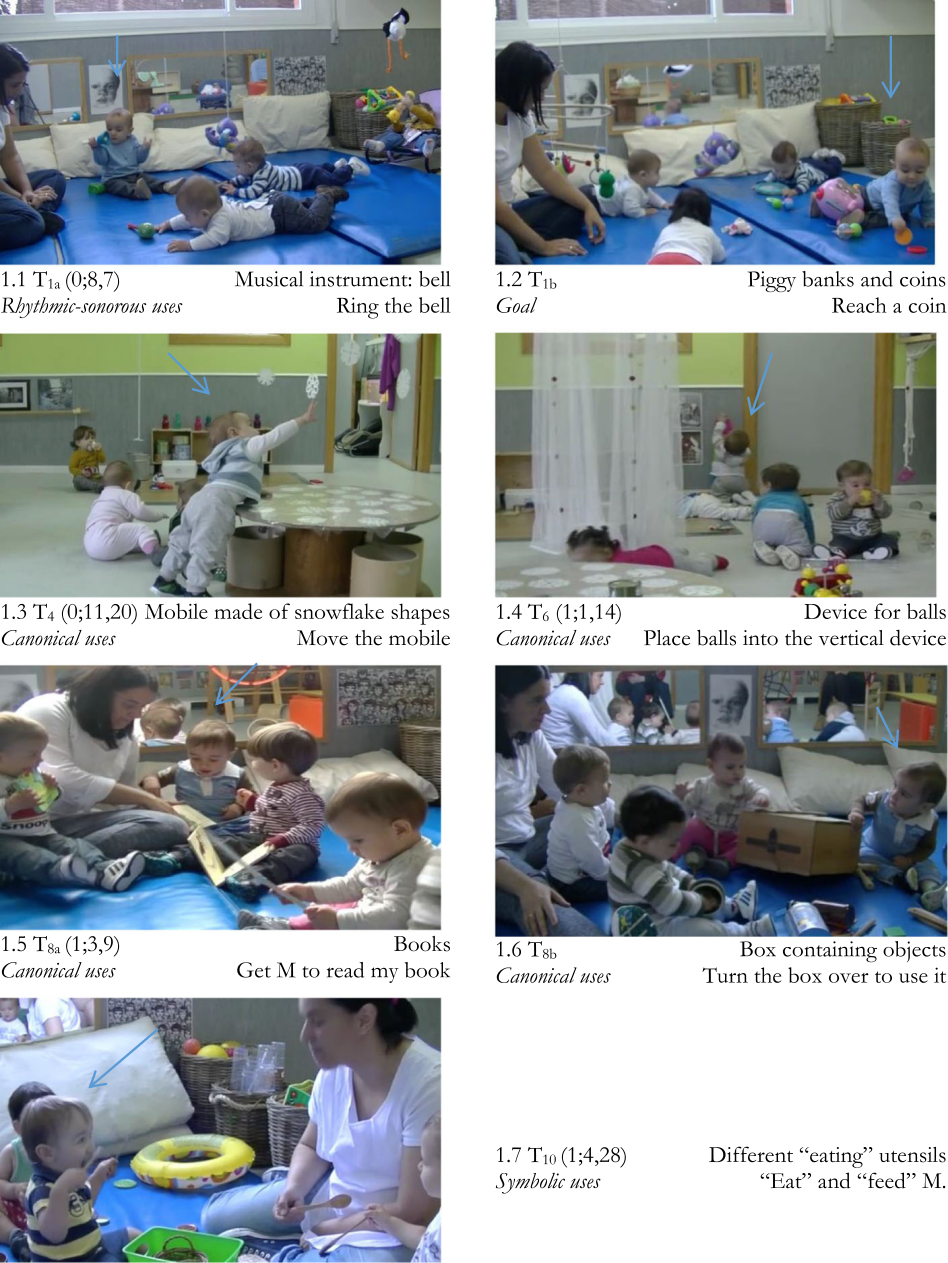
Analysed sequences T_1_-T_10_ for Ils (age). Materiality involved and Goal. The blue arrow indicates Ils

We selected and analysed the seven most significant sequences (see Fig. 1) according to the criteria established. *Duration* ranged from 1′34″ (T_8b_) to 6′50″ (T_10_). If Ils set himself the same challenge in two sequences, e.g., *how to ring a bell*, we only included the one involving the highest degree of difficulty (we selected T_1_ and discarded T_2_). Ils’s specific goals were increasingly complex (see Table 4). They always consisted of cultural uses of objects and instruments. There was predominance of *canonical* uses*.* Only in the final session did his goal consist of making *symbolic* uses.

**Table 4:** *Attention, Uses of Objects, Gestures and Speech in relation to the goals in sequences T1-T10*

			T1a Bell (0;8,7)	T1b Plastic coins (0;8,7)	T4 Snowflakes (0;11,20)	T6 Balls (1;1,14)	T8a Books (1;3,9)	T8 Box (1;3,9)	T10 “Eat” (1;4,28)
			Ils self-regulates alone				Ils requests M’s intervention		
Sequence total duration			2′42”	4′46”	6′19”	2′50”	3′35”	1′34”	6′50”
Attention Objective		Duration	2′19”	2′19”	4′48”	2′50”	3′24”	1′34”	5′06”
		% of time	88.89%	48.60%	75.99%	100%	94.88%	100%	74.63%
Use-Goal		UCRS	x						
		UC		x	x	x	x	x*	
		USI							x*
Gestures	Private	Reaching	x	x			x		x
		Ostension	x	x	x				
		Touch pointing			x				
		Symbolic				x	x		
	Directed to M	Ostensive					x		x
		Touch pointing					x		x
		Symbolic					x		x
Speech							x		

**Note. Uses:** UCRS Rhythmic-sonorous canonical; UC Canonical; USI Symbolic with instrument

*Both *uses* (UC and US) were accompanied by a *gaze* directed to M with an interrogative function

At recording time T_1_ (0;8,7), one of the aims was how to ring a bell – *rhythmic-sonorous canonical uses –* (Fig. 1.1). The other aim was how to grasp a toy coin that was out of reach (Fig. 1.2). This was considered to be a cultural use because the toy coin is part of a complex object – a piggy bank used for putting in and taking out coins, and Ils already knew this object. In T_4_ (0;11,20) his aim was how to reach a mobile hanging from the ceiling (Fig. 1.3). In T_6_ (1;1,14) his aim was to place balls into a device (Fig. 1.4); he involved M in his action only once. In the last three sequences, Ils involved M in his action plan by means of looks, gestures and using objects, though he always maintained control of the situation: it was he who decided when and what M should do. In T_8_ (1;3,9) Ils used various strategies *to get* M to read to him the book he was holding between his legs (Fig. 1.5) and sought M’s confirmation before turning over and using a box (Fig. 1.6). In T_10_, (1;4,28) Ils’s goal was to perform *symbolic uses*: “eat” and “feed M”, for which he organised a complex scenario with various instruments (Fig. 1.7). He never involved M either in *non-canonical uses* or in *hints of non-canonical uses*. He performed the non-canonical uses on his own.

Ils always accompanied his activity with *gestures*, either self-directed or directed to M, of varying semiotic complexity (ostensive, pointing and symbolic).

### Attention to the Goal (on) and Away from the Goal (off)

**Fig. 2:**
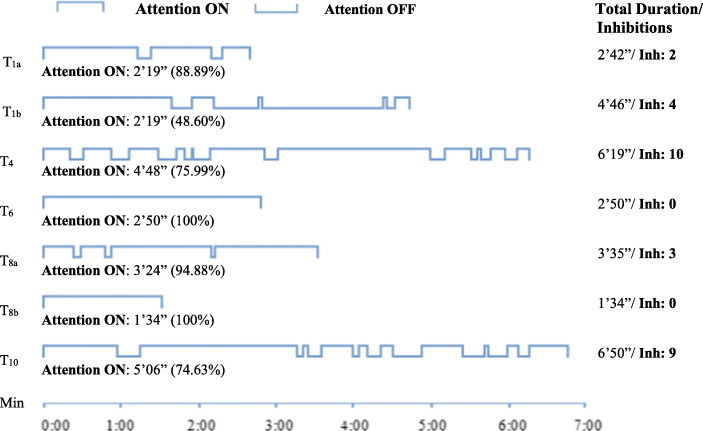
Attention ON to the objective; Attention OFF and Inhibitions in the seven selected sequences

In general, Ils alternates attention ON-OFF (see Fig. 2). When his attention is ON, he persists in his goal-directed action. When attention is OFF, he is distracted from his goal and takes interest in what is happening around him. We assumed that *inhibition* of distractors is activated every time he goes from Attention OFF to ON. Attention ON is 100% – he is never distracted from his goal – in T_6_ and T_8b_, consisting in placing balls into a device and turning over a wooden box to use it. The lowest time for Attention ON, 48.60% of total time, occurs in T_1b_, when he tries to reach the plastic coin (with 4 inhibitions). In T_1a_ and T_8a_, he is only distracted momentarily, and Attention ON predominates (with 2 and 3 inhibitions, respectively). Alternating Attention ON - OFF occurs mainly in the longer sequences, T_4_ and T_10_ (with 10 and 9 inhibitions respectively).

There is a noticeable contrast in sequence T_1_. When Ils rings the bell, Attention is ON for 88.89% of the duration of the sequence, while when he is trying to reach the plastic coin, Attention is only ON for 48.60% of the time. This difference within the same session may indicate that the materiality and degree of difficulty of the goal should be considered carefully when analysing Attention.

### Microgenetic Analyses

To illustrate the possible evolution of Ils’s challenges and specific executive processes, we performed microgenetic analysis of three sequences: T_1_ (0;8,7) (see Fig. 1.1), T_6_ (1;1,14) (see Fig. 1.4) and last session, T_10_ (1;4,28) (see Fig. 1.7), in which the goals involved three types of uses of objects and instruments.

**Fig. 3:**
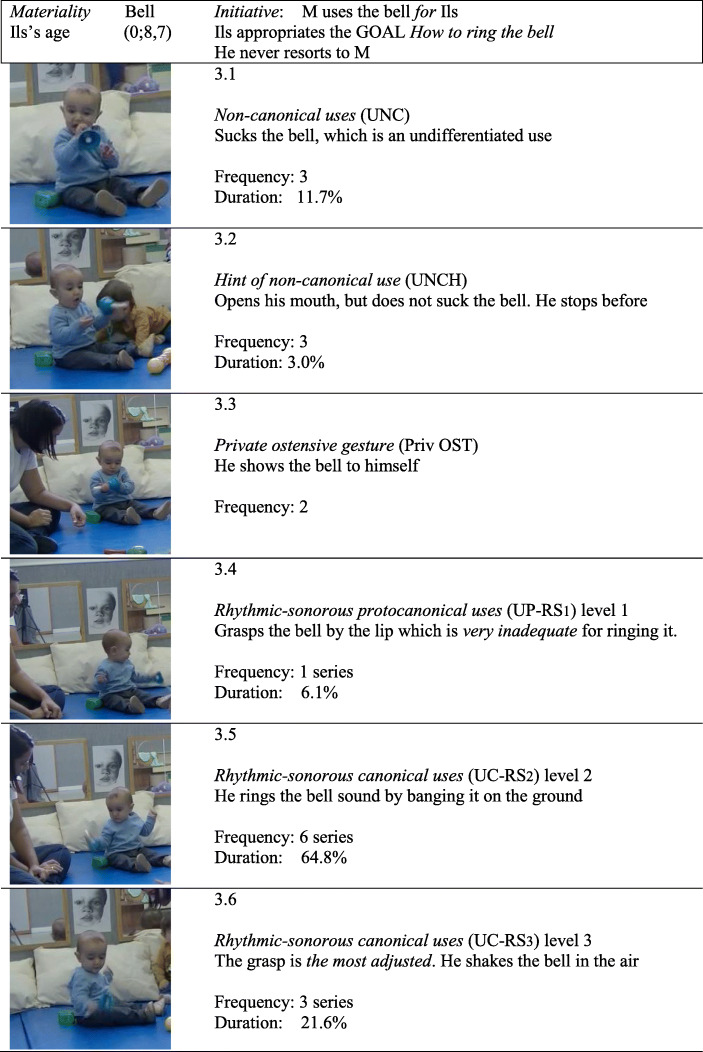
Illustrations of categories for how to ring the bell. Frequency and Duration of uses

*T*_*1*_*. Rhythmic-sonorous goal: How to ring the bell.* M *places* different musical instruments on the mat within reach of the children. Then she rings the bell *for* Ils (*Rhythmic-sonorous distant demonstration*), challenging him. Ils (0;8,7) looks interested and then *sets himself the goal* proposed by M. It is not easy because, as he does not yet control grasping, he fluctuates between types of uses that adjust to a greater or lesser degree (see Figs. 1.1 and 3).

**Fig. 4:**
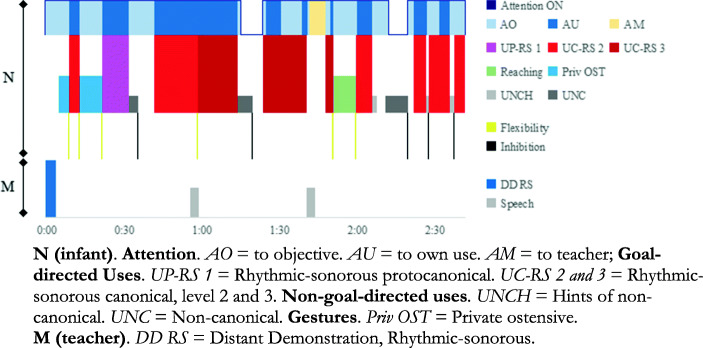
Microgenesis Ils: How to ring the bell. M: interventions. T1 Dur. 2′42″

The *goal* directs and structures his intentional interventions thanks to the functional knowledge that Ils already has regarding the bell: “it’s for ringing”. It poses a challenge because Ils does not have a clear grasping strategy. The first thing he does after grasping the bell is a *private ostensive gesture* (see Fig. 3.3), he shows the bell to himself. Next, he performs a *canonical rhythmic-sonorous use*, level 2 with intermediate efficacy (see Fig. 3.5): he grasps the bell by the handle and bangs it on the floor, while attentively *watching his own use*. Although instances of watching his own use occur throughout the sequence, they happen mainly at the beginning. He only makes a *rhythmic-sonorous protocanonical use* when he grasps the bell by the lip (level 1) with low efficacy (see Fig. 3.4). The most frequent are level 2 canonical uses, with intermediate efficacy. He also performs more efficacious level 3 uses. On isolated occasions he performs non-canonical uses (see Fig. 3.1) and hints of non-canonical uses (see Fig. 3.2). Fig. 4 shows the microgenetic analysis of the *process* followed by Ils in real time, as well as M’s interventions.

Cognitive *flexibility* is shown six times (see Table 5), through changes in the three types of uses of the bell, from use to gesture and from gesture to use, including private gestures followed by rhythmic-sonorous uses. Five times he *inhibits* uses that are not relevant to his goal (non-canonical and hints of non-canonical uses). Inhibition of *Attention* OFF, to return to his objective, occurs twice. *Attention* ON takes up most of the sequence (88.89% of total time, see Figs. 2 and 4). Above all, he attentively watches his private ostensive gestures and own uses, monitoring/evaluating them on-line. M only speaks to him to warn him of the danger of shaking the bell, saying “gently”, when Ils brushes it against his forehead. Ils then returns to safer uses of intermediate efficacy (level 2) and gives up the more efficacious (level 3) – but more dangerous – grasp.

**Table 5:** *Frequencies of Inhibition and Flexibility*

*Inhibition*		*Flexibility*	
Attention Off	2	Use to Use	1
Non-relevant uses	5	Use to Gesture	2
		Gesture to Use	3
Total	7	Total	6

**Fig. 5:**
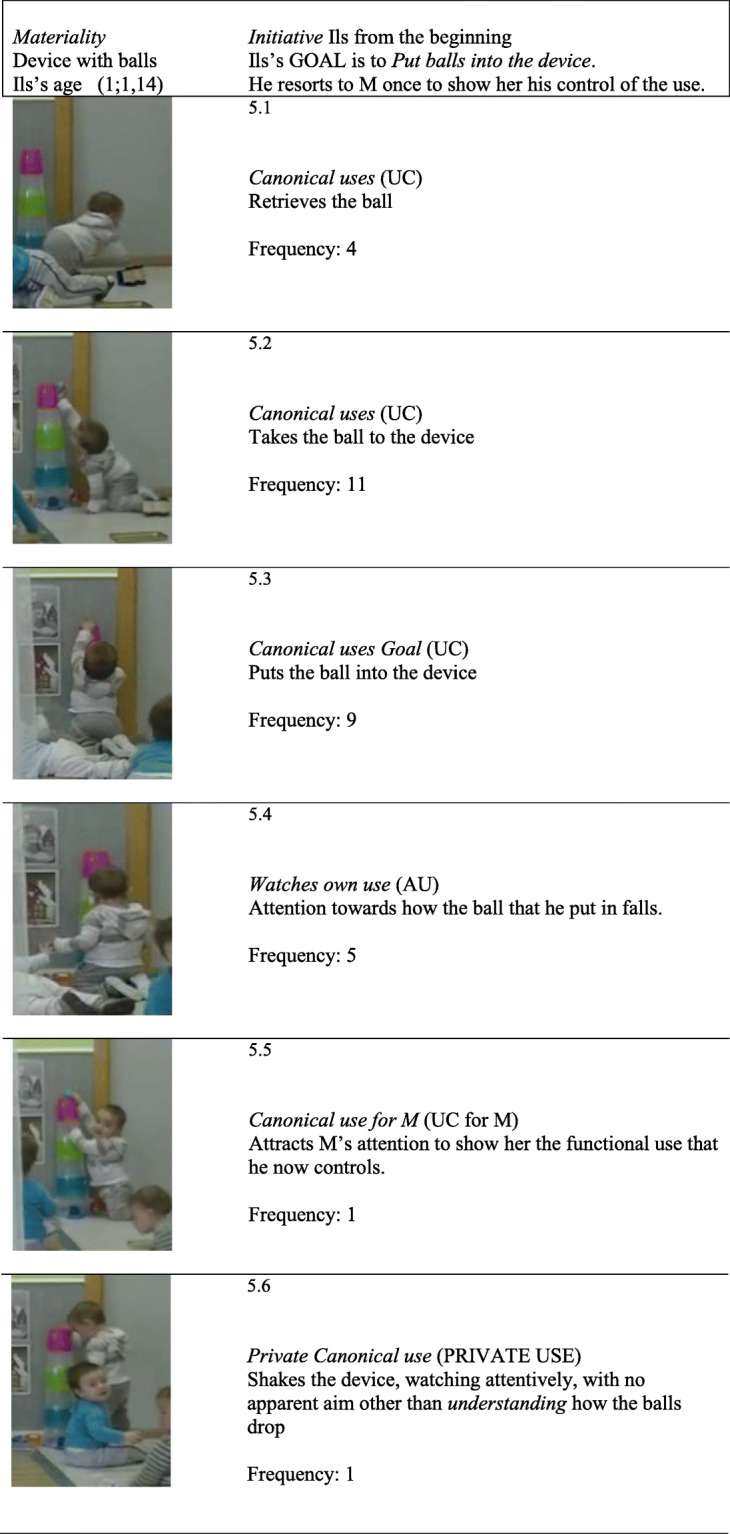
*Illustrations of the specific categories* how to put balls into the device

*T*_*6*_ *Canonical goal: placing balls into a device.* Sequence T_6_ (1;1,14) begins when Ils takes the initiative and sets himself the *goal* of placing balls in a vertical, transparent device with 6 levels (see Fig. 1.4) which allows him to see the balls dropping from one level down to the next until they come out, similar to the “gate system” used by Cerchiaro (2014) with 25-month-olds. It is the beginning of a series of uses that Ils perfects. The specific categories are shown in Fig. 5.

**Fig. 6:**
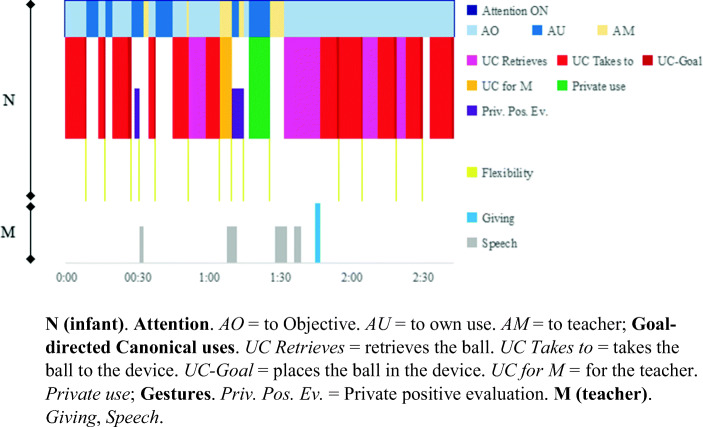
Microgenesis Ils: How to introduce balls into the device. M: interventions. T_6_ Dur. 2′50″

Like in the first sequence with the bell, here too, the *goal* directs and structures Ils’s intentional interventions, thanks to his functional knowledge of the device: “balls are put through the top”. During the first half of the sequence, every time he puts in a ball (Fig. 5.3), he attentively watches it dropping down (Fig. 5.4). After successfully putting in balls several times, he attracts M’s attention to show her “what he knows how to do now” (Fig. 5.5). He knows what he is able to do and he shares it with M. M evaluates his achievement positively by clapping. Then Ils performs a “peculiar” use that is not efficacious: he shakes the device while watching attentively (Fig. 5.6), with no apparent objective other than *understanding* how it works. The attentive gaze (Fig. 5.4) disappears in the rest of the sequence. It seems to indicate that “it is not necessary because he has understood how it works”. Indeed, the uses he performs up to the end of the sequence become more precise, “more automatic”. The only two private gestures of *positive evaluation* of his achievements occurred during the first half of the sequence. Fig. 6 shows the microgenetic analysis the *process* followed by Ils in real time.

M intervenes in isolated instances through *speech* when she *evaluates* Ils’s achievements, and by *giving-sending* the ball for him to continue.

*Cognitive flexibility* (see Table 6) is shown 14 times through changes among canonical uses, consisting of restarting the use after completing it, and retrieving the ball if it rolls away (see Fig. 5.1 and 5.2) including the one directed to M for her evaluation. There are also changes from use to gesture, when, after successfully completing two functional uses, he claps twice privately. Finally, cognitive flexibility occurs upon changing from gesture (private clapping at his own achievements) to use (returning to his objective). In contrast to T_1_, all uses are directed to the goal he has set himself, so he never *inhibits* non-relevant uses. *Attention* is always ON (2′50″, see Figs. 2 and 6), so neither does he inhibit potential distractors.

**Table 6:** *Frequency of Inhibition and Flexibility*

*Inhibition*		*Flexibility*	
Attention Off	0	Use to Use	10
Non-relevant uses	0	Use to Gesture	2
		Gesture to Use	2
Total	0	Total	14

**Fig. 7:**
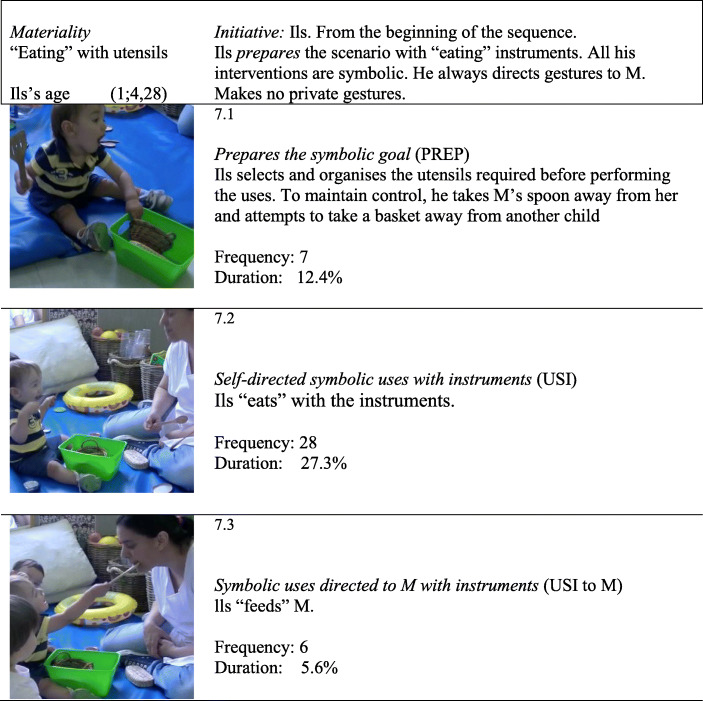
T_10_ Illustrations of the categories “eating” and “feeding” M with kitchen utensils

*T*_*10.*_ *Symbolic Goal: “Eating” and “feeding” M.* The sequence begins when Ils moves around the room collecting various utensils (see Fig. 1.7): a basket, a plastic container, spoons, ladles, which provide the basic material with which he performs the symbolic activity *afterwards*. This *planning* process takes time because the utensils are scattered around the classroom. The specific categories are shown in Fig. 7.

**Fig. 8:**
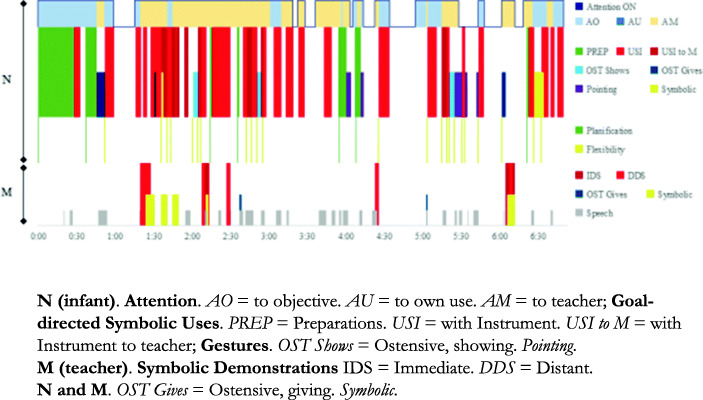
*Microgenesis Ils: How to “eat” and “feed” M. M: Interventions. T10 Dur. 6′50″*

As in the previous sequences, the *goal* directs and structures Ils’s intentional interventions. Sometimes he performs the symbols himself (Fig. 7.2), and other times he involves M (Fig. 7.3). Considering the *process* leading to the *goal*, Ils knows what he has to do right from the beginning. In contrast to the two previous sequences analysed in microgenetic detail, Ils now systematically involves M in his action plan. He alternates self-directed symbolic uses with those directed to M. He also directs to M ostensive gestures, pointing gestures and symbolic gestures. He does not make private gestures or private uses. Neither does he resort to speech here to self-regulate. Fig. 8 shows the microgenetic analysis of the *process* followed by Ils in real time.

*Cognitive flexibility* (Table 7) is shown 23 times between uses, nine altogether, when Ils alternates self-directed symbolic uses with those directed to M and vice versa. There is also flexibility between gesture and use and vice versa 13 times. As in T6, no *inhibitions* of uses are identified because all uses are relevant to his goal. However, we do identify 9 inhibitions of Attention distractors. During 5′06″ (74.63% of the total duration of the sequence) *attention* is ON (see Figs. 2 and 8) *goal-*directed, often also including M.

**Table 7:** *Frequency of Planning, Inhibition and Flexibility*

*Planning*		*Inhibition*		*Flexibility*	
Before the sequence	2	Attention Off	9	Use to Use	9
		Non-relevant uses	0	Use to Gesture	6
During the sequence	5			Gesture to Use	7
Total	7	Total	9	Total	23

M intervenes much more than in the previous sequences. However, this seems to be because it is Ils who involves M in his goal-directed action, and M responds to his demand. She does so through *Distant Symbolic Demonstrations with instruments* when she pretends to eat, and some *Immediate* ones when she “feeds” Ils, involving him in the symbol. She sometimes accompanies with *symbolic* gestures for “eating”. Twice, she gives the spoon to Ils. Again, speech (about the child’s activity) is the most frequent semiotic system in M’s communication.

## Discussion

In this article we have supported the need for *a pragmatic turn in the study of EFs*. The case study in the nursery school, classroom 0–1, showed that Ils set himself significant goals and acted intentionally from the first session when he was 8 months and 7 days old. Various executive functions were identified when trying to achieve the goal that he had set for himself. The first of these was his own goal. From this, he acted in a flexible way if we consider the changes of uses that he produced, as well as the diversity of gestures to help himself. He also inhibited inappropriate uses that diverted him from his goal. His attention was sustained, alternating attention ON, directed towards the goal, with moments of distraction, attention OFF, to return again to attention ON. In the last session, at 1;4,28, he was able to plan and prepare the necessary materials in order to realize a symbolic scenario in which he repeatedly involved his teacher. Next, a detailed discussion of the four research questions as well as the executive components identified in this research is presented.

### Research Questions

1. The first question was whether, during everyday classroom activities, Ils set himself *significant goals* which, due to their difficulty, posed a challenge. This itself would involve the presence of EF. Results show that from the first session, at age *8 months, Ils already set himself goals* involving *significant challenges*, and such challenges were seen in all sessions and required considerable effort on the plane of action. Only once – sequence one in session one – did M take the initiative, but Ils immediately made the challenge his own. In the rest of the sequences, it was Ils who determined what the challenges were, the pace of his action, and when to begin and/or end. He made use of the materiality within his reach, which he used *when* and *how* he wanted. The ultimate responsibility in the choice of challenges was always the child’s.
2. The second research question was: *what did* the challenges *consist of and how did they evolve*? It was found that the challenges the child set himself from 8 to 17 months of age always consisted of performing *cultural uses* of *objects* and *instruments*. These are public uses, subject to standards shared by the community of users.
3. The earliest, most basic uses, at age 8 months, were *rhythmic-sonorous canonical uses,* when Ils’s goal was to ring a bell. This was complex because he resorted to three ways of holding it, which substantially altered the sound produced. In the same session, he set himself another challenge of how to reach a plastic coin that was out of his reach with the help of another plastic coin. This was instrumental use at age 8 months! No doubt it was one of the earliest manifestations of use of an instrument (Piaget 1936/1965) to reach something. The particularity is that the object pursued (the plastic coin) formed part of a complex object known to Ils: the plastic coins were used for putting into and taking out of a piggy bank. His interest in reaching the plastic coin seems to be framed within a broader understanding of the function of the complete object.
4. The following *goals* involved functional *canonical uses*. In T_4_, at age 11 months, his goal was to shake a mobile made of paper snowflakes hanging from the ceiling. The difficulty was that since he could only remain in a standing position with great effort and could not easily reach the snowflakes, he had to lean on a table strategically placed by the teacher beneath the mobile (see Fig. 1.3). In T_6_, at age 13 months, the complexity of the challenge increased substantially. It consisted of placing balls into a vertical device. Here, as shown, he controlled functional usage from the beginning. What is noteworthy in this sequence is that the purpose of some of Ils’s behaviours did not seem to be directed towards success, but rather at *understanding* why and *how* the device enabling balls to tumble down worked. Thus, he directed a series of *attentive gazes* to the *trajectory* of the ball *as it fell* from one level to the next. On one occasion, he also watched the device attentively *while shaking it*. Indeed, the attentive gazes disappeared during the second half of the sequence, coinciding with the increase in efficacy of use. It may illustrate the difference mentioned by Piaget (1974) between “*reussir et comprendre*” – performing a task and understanding it. All of which is similar to what Piaget refers to in relation to the behavior of one of his children:

[O]ne of my children was in his park (he was in the sensory-motor stage, that is, before language) and I held an object up to him in a horizontal position, so that if he tried to attract it to himself, the bars of the park would prevent him from doing so. He tried all sorts of positions until he finally succeeded. But he had succeeded by chance and this did not convince him. He took the object out of the park and started again, until he understood how to guide it through the bars. *Success was not enough: he was only satisfied when he understood how it worked*” (Piaget 1973/1977, p. 113, emphasis added).

Another novelty in sequence in T_6_ is that Ils once involved M in his action plan by explicitly showing her the use that he was *going to perform,* in the knowledge that he could by then control it. He expected M’s positive evaluation of the functional use that he controlled and obtained it in the form of applause.

In the two following sequences, in T_8_ at age 15 months, Ils’s goals were also related to *functional uses* of the materiality (objects and instruments) available to him. The novelty was that he always involved M in his goals. Once he insisted repeatedly and in various ways that M “read” him the book he had chosen. This involved various strategies, because M could not be available exclusively to Ils (see Fig. 1.5). In the same session, he tried to turn over a wooden box in order to fill it with several utensils, involving M by looking at her questioningly, “asking for permission” (see Fig. 1.6).

2.3 The final session, T_10_, at age 17 months, provided further novelties. The first and most noticeable was the increase in the semiotic complexity of the challenge Ils set himself, which consisted of making *symbolic uses.* He used *kitchen utensils*. Before using them, he *prepared* by collecting several containers, spoons, ladles, etc. from around the classroom. It can be said to have *planned* his action *before* performing it. Moreover, he systematically involved M, but always while maintaining the control and initiative. It was he who determined when and how M should act. It can thus be confirmed that behaviours in which a child resorts to others can be considered self-regulatory, if it is the child who controls exactly what he/she requests and expects from the other person (Basilio and Rodríguez 2011). We considered that a symbolic sequence could show an executive function, in accordance with the thesis of Barker and Munakata (2015), when, based on Vygotsky, they claim that “advanced forms of pretend play allow children to practice, generating, maintaining, and carrying out plans and objectives.” (p. 95) (see also, Elias and Berk 2002; Bodrova et al. 2013). The situation presented here fulfils these characteristics.

To conclude, it can be said that the diversity of goals that Ils set himself from the first session (0;8,7) to the last session (1;4,28) seem to show that there was evolution. The simpler goals were set at the beginning, T_1_, (rhythmic-sonorous canonical uses), while the most complex goal (symbolic uses, both self-directed and directed to M) occurred in the last session, T_10_, and included a range of preparation and planning. The goals related to functional uses were the most frequent, with different degrees of complexity in the rest of the sequences.

3 The question ‘What *means* did Ils employ to attain his goals and how did they *evolve*?’ was answered by looking at his gestures, which seemed to fulfil an important role in achieving goals. Obviously, the gestures were never the goals, but they did play an important part in attaining them. There was also an interesting interplay between uses and gestures, which were closely articulated.

We also observed a progression in the semiotic complexity of gestures, such as those with uses of objects and instruments (see Table 4). In the two first sequences, in T_1_ (0;8,7) there were only *private ostensive gestures* when Ils repeatedly showed himself the bell and the plastic coins before using them. Bell and coin are sign and referent at the same time. He *showed* himself those objects to *think about* those objects. He showed them to himself repeatedly while figuring out how to ring the bell or reach the other plastic coin. In T4 (0;11,20) he also made private ostensive gestures with the snowflakes. He showed them to himself to understand, and for the first time, produced *pointing gestures touching* the referent.

In T_6_ (1;1,14), he only produced *private symbolic* gestures when he applauded his own success on two occasions upon performing the canonical use of placing the ball in the device. In T8a, at age 15 months, he only produced one private gesture, symbolic of self-evaluation, directing the rest to M: he made ostensions of the book for M, also pointing/touching the pictures in the book and communicating to M through symbolic gestures. These were all part of his strategy to get M to “read” the book. It was here that he produced the only word found, “all done”, directed to M. During the same session, in T8b, where his aim was to turn over the box, although he did communicate with M, he did not use any gestures. This indicates that whether or not gestures are used depends on the child’s development, but also on the materiality involved in the action plan. In the sequence of the final session, T_10_, at age 17 months, no private gesture was produced. All of Ils’s gestures - ostensive gesture of the spoon to show and give, pointing gestures, and symbolic gestures of “eating” - were directed to M.

These data are in agreement with the studies that suggest that private gestures may have a self-regulatory function. One of the star gestures has been pointing in its attentional function (Delgado et al. 2009, 2011; Carpendale and Carpendale 2010). Here, however, it is seen that private ostensive gestures were the first gestures whose function served self-regulation, in agreement with the results of previous studies in the home when children find difficulties in using complex objects according to their function (Basilio and Rodríguez 2017; Moro 2012; Moro and Rodríguez 2005; Rodríguez and Palacios 2007) and at nursery school, classroom 0–1 (Rodríguez et al. 2017b; Guevara et al. 2020).

To conclude with research questions 2 and 3 concerning the development of goals and of the means to attain them, it appears that the challenges that Ils set himself changed substantially over the sequences in terms of the complexity of the semiotic systems employed, which were basically non-linguistic. It was to be expected that in T_1_ at age 8 months he would not set himself challenges involving management of symbols, but that in T_10_ at 17 months, he would. And that is precisely what occurred. The same is true of gestures. His first gestures – ostensive gestures – were semiotically less complex. However, during the final session, there were many gestures, ranging from the most basic, such as ostensive gestures, to the most complex, such as the pointing gesture or symbolic gestures.

4 The final research question concerned the teacher’s educational action. M’s interventions were basically suggestions made by *placing the material within reach* of the children. M’s participation was always in the *background*, observing the children and *giving them time*. This suggests that the choice of material plays a very important part in the child setting himself challenges. It should have an adequate level of difficulty and should be within the child’s zone of proximal development.

Only in isolated instances did M perform *Distant Demonstrations,* but *Immediate Demonstrations* – involving the child in the use – occurred when Ils directly requested M’s intervention. She also communicated by means of *ostensive gestures*, by giving him objects, and by means of *symbolic* gestures. Her most frequently used semiotic system was *Speech.* She suggested caution, gave positive evaluation of some achievement or commented on the course of action of Ils, but her speech was always produced as accompaniment to the child’s action, never as instructions for what he should or should not do. In short, M’s contribution enabled Ils to set himself meaningful challenges. It consisted of (i) making available *material* objects and instruments that were particularly *appropriate* to the child’s level of development, (ii) *stepping back and not intervening* unless needed or because the child explicitly requested her to do so, (iii) giving the child enough *time* to set his own challenges, and to err and to correct himself, (iv) indicating her *presence* in some way (through looks, positive evaluation, isolated comments, etc.) (see also Belza et al. 2019).

To conclude, it is in M’s action that we find one of the most important differences from the role played by the experimenter in classical standardised EF tasks in which the initiative is the experimenter’s. It is the experimenter who tells the child what the *goal* is and what, when and how to do things. It is assumed that this becomes a genuine challenge to the child. The risk is that if it does not, then the EFs and the tasks used for studying EFs would be moving along parallel paths. Moreover, “cleanliness of tasks” is prioritised, to the detriment of any meaningfulness that the task might have from the child’s standpoint.

### EF Components Studied: Own Goal, Attention, Cognitive Flexibility, Inhibition and Planning

The following EF components were analysed in this study: Goal, Attention, Flexibility, Inhibition and Planning.

Due to its difficulty, a *Goal* poses a challenge. Ils’s goals were always related to the performance of cultural uses of objects and/or instruments. As mentioned above, the earliest consisted of achieving rhythmic-sonorous uses, and the rest were canonical, with the last consisting of performing symbolic uses.

With regard to *Attention*, which was always considered in relation to the goals that the child had set himself, we distinguished between (i) Attention ON directed to the goal or the means to achieve it, and (ii) Attention OFF when attention deviated from the goal or the means, and was directed to ongoing events in the classroom. As shown in Fig. 2, the percentages of Attention ON ranged from 48.6% of the total sequence time to 100% in two sequences in which Ils was constantly attentive to his own action in relation to the goal he had set himself.

The *Cognitive Flexibility* required to achieve the goals was shown in all sequences through non-linguistic *semiotic systems*, which included uses of objects, instruments and gestures. Self-regulation with language occurred only in T_8a_ (1;3,9) “all done”, and in T_10_ (1;4,28) when he produced some vocalisations. Changes between uses, between gestures, from gesture to use or from use to gesture were considered to be indicators of flexibility. The most frequent were between uses. This means that the child performed significant actions from various semiotic systems, considering the problems he had to resolve according to the goal he had set himself. All the changes, which often affected the means, were selected and performed by the child. They increased noticeably between the first session and the last, as shown in the three microgenetic analyses in T_1_, T_6_ and T_10_ (Figs. 4, 6 and 8).

*Inhibition* was considered in relation to *uses* and *attention*. Regarding uses, inhibition took place when uses that diverged from the goal, such as sucking the bell (see Fig. 3.1), stopped in favour of others that did lead to the goal, such as ringing the bell. Inhibition of non-relevant uses only occurred in the first sequence analysed in microgenetic detail (see Fig. 3.2). It never occurred in the other two sequences because uses were always functional and in accordance with the goal, even though efficacy could vary. Inhibition was also considered in relation to *Attention* when Ils inhibited Attention OFF that diverged from his goal, to return to attention ON related to the goal or the means to achieve it. Ils inhibited Attention OFF in most sequences. In the two sequences in which he was never distracted from his goal (T_6_ and T_8b_), Attention was always ON, and there was never an inhibition of Attention OFF. This occurred in the second sequence (T_6_) analysed in microgenetic detail. The results found here in relation to inhibition are not consistent with one of the most prevalent ideas in the literature about inhibition, according to which, in the *Dimensional Change Card Sort* at age three, children cannot inhibit the current perceptive dimension in favour of a different one (colour and shape, for example) (Capilla 2007; Diamond 2002). It could be hypothesised that it is easier to inhibit an inadequate use or Attention OFF when the child has set himself/herself a significant goal than when – as happens in standardized tasks – something that was initially correct becomes incorrect.

*Planning* only occurred in the last sequence, at age 17 months. Ils clearly prepared his scenario by gathering, organising and arranging the material that was scattered about the classroom *before* making the first symbolic use of it. It was the material, classified and organised according to his goal, which served as the material basis on which to construct the symbolic sequence. Prior to this sequence, it cannot be stated that Ils planned his action in advance. In the previous sequences, things seemed to happen “on-line”, based on his knowledge of the uses of the objects and instruments involved, but nothing in his action suggested prior planning.

## Conclusion

Taking into account the various critical voices, including the case study presented here, it appears necessary to introduce a pragmatic turn in the research on the origin and early development of executive functioning: a change of paradigm which considers children as agents, with their own initiative. A theory that aims to explain the origin of cognitive control from the end of the first year of life, must necessarily analyze what significant objectives children set for themselves, and what they do to achieve them. It must also study how challenges evolve and how the means employed become more adjusted. On the other hand, it also has to consider the ecological validity of the situations studied in its socio-cultural niches. This implies studying the everyday situations in which children are found and taking seriously their level of socio-cognitive development; that is, considering what they can do without taking for granted skills they are still far from acquiring, or that are in a very early stage of development. Another aspect of ecological validity relates to the characteristics of the materiality involved. In everyday life, objects (and instruments) are used in pursuit of practical objectives. Segmented properties of materiality such as color, shape or size, might not be relevant to the objectives that children have when they act on the environment in their first and second year of life. Finally, the traditionally analyzed components of executive functions - flexibility, inhibition, attention, working memory, goal setting or planning - must be studied within an integrated view of the child’s activity. This involves avoiding the traditional split between components and considering their dynamic articulation when the child tries to solve the problem at hand.

In the case study presented, these aspects were considered. Thus, the starting point was the everyday situation of the classroom. That is, the educational situation designed by the teacher with the materiality made available to the children, without any modification.

The focus was set on the child, as an active and intentional subject who set his own significant challenges and who does not merely react passively to “stimuli”. This is a novelty compared to other studies, since no verbal instructions were given as to what goal the child should achieve. The child’s intentionally produced actions and gestures came into the spotlight. With the action, the materiality involved (objects and instruments) also became very relevant. The specific objectives he gave himself varied according to the objects and instruments he used each time. However, the semiotic complexity of the types of uses increased. His first goals were to make rhythmic-sonorous canonical uses. The most frequent challenges consisted of performing canonical or functional uses. The last ones to appear, at 17 months, were symbolic goals. Only at this point did he anticipate and plan his future action.

If we consider the status of *error*, it should be noted that, while in classical EF tasks, children do or do not commit errors according to the task set by the experimenter, in the everyday situations shown in the current study, errors, strictly speaking, cannot occur. There is, in contrast, a range of possible uses which may be more or less efficacious. For example, when the child is seeking a solution to the challenge he has set himself, and during that search makes uses that are not efficacious, those uses are not errors. A less efficacious use could enable the search for a solution to continue, or could form part of the solution later on. With regard to error status, it may be said that the difference between the classical tasks set by the experimenter and the child’s active search to resolve a challenge do not seem to follow the same path.

Ils almost always took the initiative. Exceptionally, when it was his teacher’s initiative, the child took up the challenge and made it his own. He also showed persistent behavior. If at first he did not get results, he tried again and again. To achieve his goals, he always used adequate means. He did it in a flexible way, adjusting his behavior to the results of his own action. He was able to recognize his achievements and to inhibit inadequate behaviors - for example, non-canonical uses which took him away from the goal he set for himself. Gestures, especially private ones, helped him to resolve his difficulties. As happened with uses, gestures also gained in semiotic complexity. Private ostensive gestures were the first to appear. Their leading role in the processes of self-regulation is confirmed. The gap between private ostensive gestures and private pointing gestures is also confirmed. Pointing gestures, being more complex, appear later when used for cognitive control. This is very striking because the pointing gesture has attracted the most attention from researchers in self-regulation studies. The direction of the child’s action also varied. Initially, he always set the challenges for himself, and searched for solutions, alone. From 13 months, he began to involve his teacher in his plan of action.

His level of attention was always very high, although there were also moments of alternation between Attention to his goal, ON, and when he deviated his attention from his objective, OFF. This fact is very relevant when analyzing everyday situations. It is adaptive to alternate attention between one’s goal and what is happening all around.

Finally, another novelty of the pragmatic turn adopted in this study consisted in not isolating the components of executive functioning. Goal setting, flexibility, inhibition, attention, and planning were analyzed within the course of the child’s intentional activity.

### Limitations of this Study and Future Lines of Research

With regard to methodology, the main limitation of this study is that it is a case study in a single school. Perhaps not all schools promote children’s self-regulation in the same way or with the same emphasis. Moreover, one video-recording per month may be insufficient to analyse the development of executive functions and self-regulation, especially at ages where significant changes occur in a very short time. Other limitations may be identified in the 0–1 classroom, e.g., the fact that the objects and instruments included in the study were those provided by the teacher. The question remains open as to whether there could be moments of cognitive control with other objects and instruments.

With regard to future lines of research, further studies need to take into account the everyday situation of children at nursery schools, institutions for early infancy in general, and at home, as well as what educational actions favour or hinder the appearance of executive functions. Further research should consider the aforementioned limitations and act accordingly. Moreover, it should explore what objects and instruments children are interested in and which they will use for setting themselves challenges. Further longitudinal studies should take into account the enormous complexity involved in children’s cognitive control at this age, and consider collecting data over shorter times. There is a need to continue to explore the relation between psychological development and executive functions with the appearance of increasingly complex challenges and more distal means, as well as what new forms of cognitive control children use with relation to new, increasingly difficult challenges.

Based on our results, new questions arise in related fields. For example, how does cognitive control originate and develop in children with atypical development? A particularly relevant case is that of children on the autistic spectrum. Such knowledge would enable possible early warning signs to be recognised and new forms of intervention to be developed. Finally, future research could analyse the role of adults in general and teachers in particular in the origin and development of EFs and self-regulation, what elements of interaction and/or educational intervention favour the development of EFs and self-regulation, and how they help children to self-regulate by progressively yielding the initiative in activities and regulation.
